# Spatiotemporal levee erosion and wavefront velocity dataset from *in-situ* wave overtopping experiments

**DOI:** 10.1038/s41597-026-07477-9

**Published:** 2026-05-29

**Authors:** Masoumeh Ebrahimi, Myron van Damme, Niels van der Vegt, André Koelewijn, Vera van Bergeijk, Claire Damblans, Bilal al Tfaily, Abdelkrim Bennabi, Ammar Aljer, Sandra Soares-Frazão

**Affiliations:** 1 Institute of Mechanics, Materials, and Civil Engineering (iMMC), UCLouvain, Louvain-la-Neuve, Belgium; 2 Rijkswaterstaat WVL, Utrecht, The Netherlands; 3 Department of Civil Engineering and Management, University of Twente, Enschede, The Netherlands; 4 HKV Consultants, Lelystad, The Netherlands; 5 Department of Flood Defence Technology, Deltares, Delft, The Netherlands; 6 Department of Coastal Structures and Waves, Deltares, Delft, The Netherlands; 7 Institut de Recherche de la Construction (IRC), ESTP-Paris, 94230 Cachan, France; 8 Laboratoire de Génie Civil et Géo-Environnement (LGCgE), ULR 4515, University of Lille, Lille, France

## Abstract

During extreme storm events, waves that run-up and overtop levees cause a significant hydraulic load on the landside slopes. In order to determine the effects of these loads on the flood risk, it is necessary to determine the sensitivity of the landside slope to erosion. Models exist for determining the erosion resistance of grass covers to wave overtopping. The challenge has been to qualitatively describe the erosion process due to wave overtopping and to quantify the erosion rate of clay exposed to wave overtopping loads. To address this challenge, prototype scale wave overtopping experiments have been performed. To facilitate the transfer of the results to other locations and clay types, the Erosion Function Apparatus has been used to predict the erosion characteristics from small samples collected from the test sites. Here we present a dataset containing measurements from four experimental campaigns designed to evaluate the erosion resistance of existing and newly constructed clay covers under controlled overtopping loads. It includes soil properties, hydraulic loading programs, overtopping characteristics, wavefront velocities, and erosion progression data. The dataset enables comparative analyses across levee types and provides a reproducible foundation for model calibration, erosion assessment, and flood defence design.

## Background & Summary

**Fig. 1:**
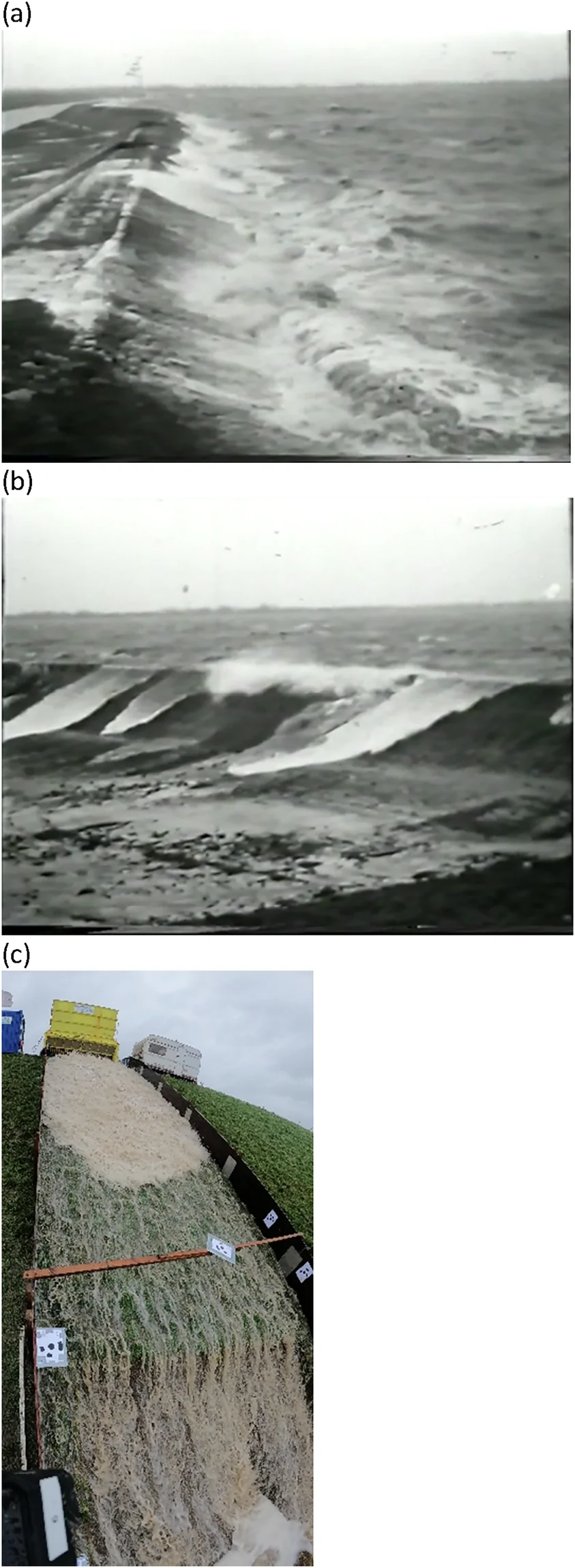
Visualization of wave overtopping on levees under real and simulated conditions: (**a**) real wave overtopping during a storm event near Wilhelmshaven, Germany, in 1954 (seaward view along the North Sea); (**b**) landward view of the same 1954 event; and (**c**) simulated wave overtopping generated using the Wave Overtopping Simulator.

In addition to rising mean sea levels, climate change has the potential to intensify storms and extreme weather events, increasing the likelihood of extreme loading conditions on earthen embankments, such as dikes and levees. These hydraulic loads can manifest as overflow —when water levels exceed the embankment crest level— or as wave overtopping, when the water level is below the crest height, but waves are high enough to run-up and overtop the dike crest, or as a combination of both. Figure 1a,b illustrate an example of a levee wave overtopping event along the North Sea, recorded during a storm near Wilhelmshaven, Germany, in 1954. To assess flood risk, it is crucial for levee managers to understand the ability of these covers to sufficiently withstand these extreme conditions. To this end, the data presented in this paper was assembled as part of the European Interreg NWE project Bonsai (bonsai.nweurope.eu) in order to evaluate the short-term resilience of levees in North-West Europe. Part of this dataset was produced and published publicly via the Data Wizard^1^ produced within the European Interreg NWE Polder2C’s project (polder2cs.eu).

Other datasets related to flood risk analyses already exist, though they primarily focus on historical post-event observations or spatial mapping. The International Levee Performance Database (ILPD)^2^ provides historical levee failure data allowing for macro-scale analysis of failure mechanisms and breach characteristics. Similarly, the recently published Levee Loading and Incident Dataset- Overtopping (LLID-OT)^3^ reports historical riverine levee overtopping events supported by data on hydraulic loading, soil and foundation classifications, construction/maintenance characteristics, geometry, and breach dimensions. Comprising a subset of 487 overtopping events from the U.S. National Levee Database (NLD), the dataset enables systemic risk assessment and data-driven breach modeling. Furthermore, OpenDELvE^4^ strengthens levee data by integrating high-resolution spatio-geometrical, overcoming previous documentation limits by mapping the often poorly documented locations and characteristics of flood-protection levees on river delta. While such databases are crucial for flood hazard modelling and mapping, they do not report the dominant physical processes at the very high spatial and temporal resolutions of individual failure events. Datasets capturing these processes, however, are of paramount importance for the development and validation of numerical models simulating a single failure event. The dataset outlined in this paper^5^ fills a critical gap by enabling high-resolution, micro-scale analysis of the erosion process under wave overtopping conditions conducted on prototype levees.

**Fig. 2:**
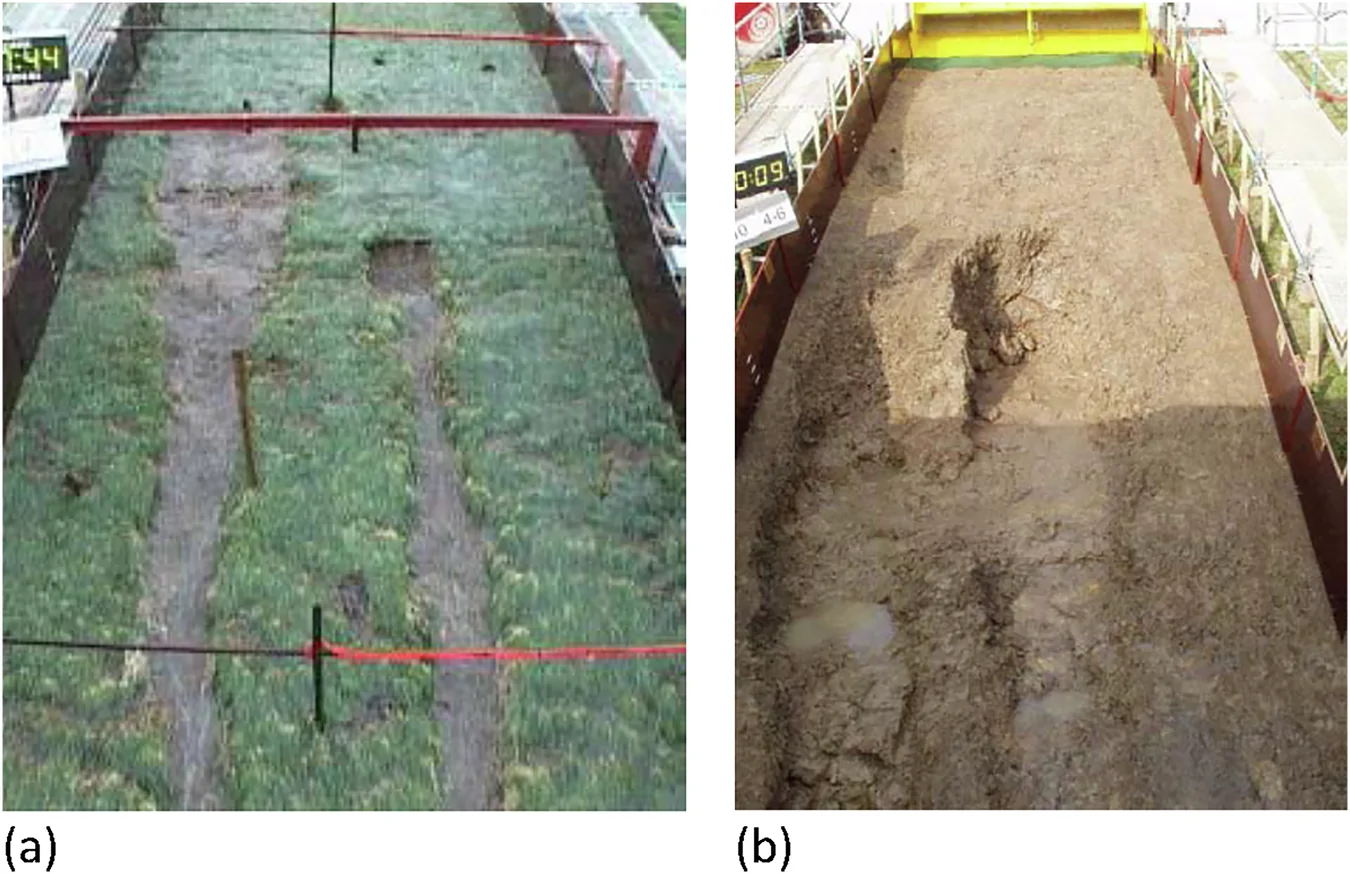
Landward erosion of (**a**) grass-covered levee and (**b**) exposed clay cover due to wave overtopping^10^.

While the erosion resistance of levee grass covers due to wave overtopping^6–9^ has been extensively studied (Fig. 2a), the spatiotemporal progression of erosion in the underlying cohesive clay soils (Fig. 2b), specifically headcut initiation and development, remains poorly quantified^10–14^. Consequently, evaluating the resilience of bare clay and innovative soil mixtures, such as lime-treated clays^13,15^, necessitates controlled prototype testing on real structures under extreme conditions before widespread application.

Differentiating itself from existing historical databases, our dataset provides paired, multi-scale data derived from highly controlled *in-situ* wave overtopping experiments (Fig. 2c) and corresponding Erosion Function Apparatus (EFA) laboratory tests^16,17^. This distinct structure offers an unprecedented, reproducible foundation specifically designed for studying scale effects between laboratory and field conditions. Ultimately, it provides the unique high-resolution morphological and hydrodynamic data required by modelers to calibrate empirical soil erodibility parameters and rigorously validate advanced numerical models for levee erosion.

The dataset outlined in this paper illustrates the challenge of correlating the outcomes of reduced size erosion tests against prototype sized tests on the same material by providing both the well-documented field data as well as the corresponding results from the tests with the EFA.

The test cases included in the dataset are: (i) Delfzijl region as part of ComCoast project, (ii) and (iii) Living Lab Hedwige-Prosperpolder (LLHPP) area within Polder2C’s and Lime in Clay projects, and (iv) the Lelystad region as part of the Erosion Resistance of Transitions project.

The tests focused on erosion of the landward slopes composed of bare clay (i.e. without a grass cover) except in case (iv) and were subjected to wave overtopping loads generated by a wave overtopping simulator. During the tests, erosion progression was measured manually and monitored through close-range photogrammetry, which generated high-resolution topographical models for detailed analysis.

In summary, this dataset provides not only insight into the erodibility of various clay soils but also offers a unique opportunity for modelers to validate and calibrate numerical models against a wide range of levee overtopping conditions. As summarized in Table 1, the core value of this dataset is defined by five key features:

- **Comprehensive Soil Characterization:** Detailed experimental setup characteristics and geotechnical properties covering diverse levee cover layers, including natural bare clay, innovative lime-treated clay, and well-compacted clay.
- **Paired Multi-Scale Testing:** A direct coupling of full-scale *in-situ* field experiments with corresponding small-scale Erosion Function Apparatus (EFA) laboratory tests conducted on undisturbed clay samples extracted from the test locations.
- **Controlled Hydraulic Loading:** Detailed time-series data of overtopping conditions, featuring specific wave volumes, frequencies, and loading patterns generated by the Wave Overtopping Simulator.
- **High-Resolution Hydrodynamics:** Precise quantification of wave overtopping hydrodynamics, featuring wavefront velocities derived from both manual (frame-by-frame) video analysis and automated video detection (Wavefront Tracker).
- **High Spatiotemporal Resolution of Morphology:** Spatiotemporal tracking of erosion progression and headcut formation, captured through high-resolution 3D topographic models derived from close-range Structure-from-Motion (SfM) photogrammetry and manual profiling

**Table 1:** Data summary.

A brief introduction to the erosion process in clay soils is essential before the methods and data presented in this paper can be fully explained.

## Erosion Mechanism and Parameters

**Fig. 3:**
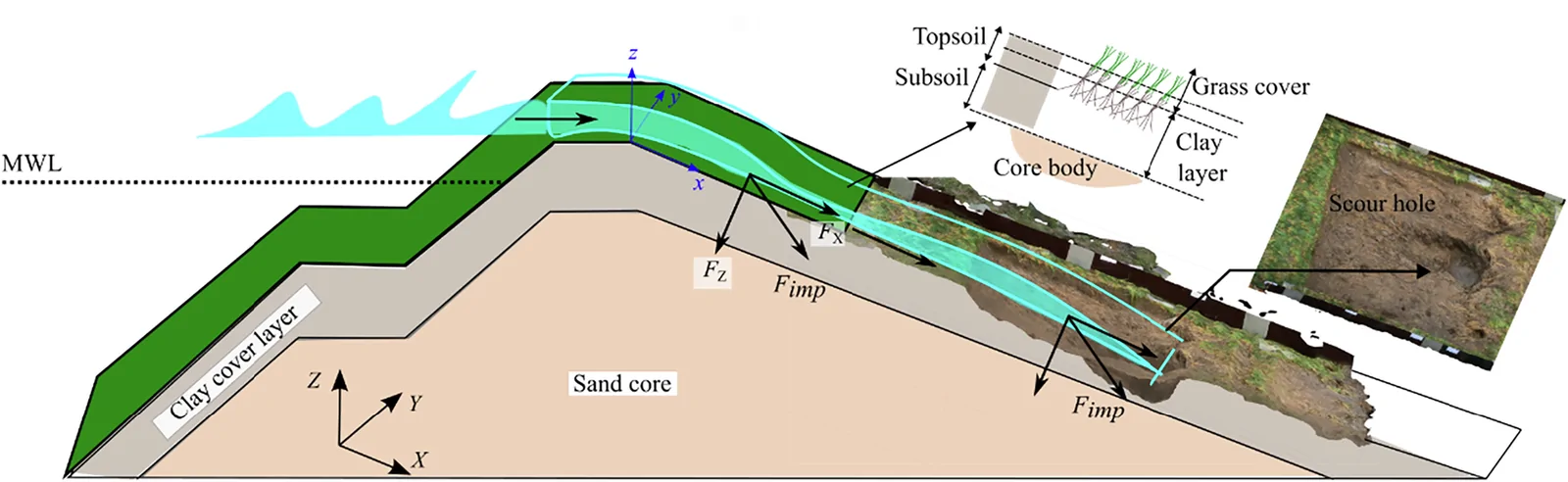
Schematic representation of levee cross-section, including the different layers and the overtopping wave with its exerted forces, as well as an example of an erosion pattern in the original clay cover.

A well-maintained grass cover gives a significant resistance to erosion. A grass cover is typically considered to have failed once erosion penetrates about 20 cm into the topsoil, indicating that the sod layer has been damaged^18^. Soil beneath the grass layer is then exposed to the overtopping flow (see Fig. 3).

In cohesive materials, the dominant form of failure is the formation of a scour hole in the levee cover as shown in Fig. 3, which subsequently develops into a headcut^19^, a steep cliff-like feature that becomes progressively steeper. This headcut then migrates toward the crest of the levee. The rate of this migration governs the whole breaching process leading to the complete failure of the structure.

The physical processes involved in erosion can be described by well-known Eq. (1)^20^:

1 $${\varepsilon }_{r}={k}_{d}{(\tau -{\tau }_{cr})}^{\alpha }$$

Where, *ε*_*r*_ is the erosion rate (m/s), *k*_*d*_ is the erodibility coefficient (m/s)/(N/m²), *α* is an empirical exponent, assumed by Hanson *et al*.^19^ to be unity, *τ* is the average hydraulic boundary shear stress (Pa), and *τ*_*cr*_ is the critical shear stress (Pa).

Erosion initiates when the applied shear stresses exceed the critical shear stress of the soil. Gradients in excess shear stress or local variations in soil erodibility induce the formation of a local scour hole. As this scour hole deepens the flow separates from the surface to form an impinging jet. This plunging flow concentrates high turbulence and hydrodynamic impact pressure at the base of drop, driving undercutting and mass failure that evolves the scour hole into a steep, vertical headcut. The speed of this headcut migration, dX/dt, can be described by Eq. (2):

2 $$\frac{dX}{dt}=\frac{T{k}_{d}(\tau -{\tau }_{cr})}{{E}_{v}}$$

where, *T* is the distance (m) of headcut displacement at each mass failure, and *E*_*v*_ is the depth of erosion on the vertical face (m) required to destabilise the headcut.

Empirical parameters in these formulas, such as the erodibility coefficient the critical shear stress should be calibrated according to the specific soil characteristics as demonstrated by previous studies^19^. The dataset provided here enables such calibration and supports the derivation of soil erodibility parameters for each tested levee.

## Methods

**Fig. 4:**
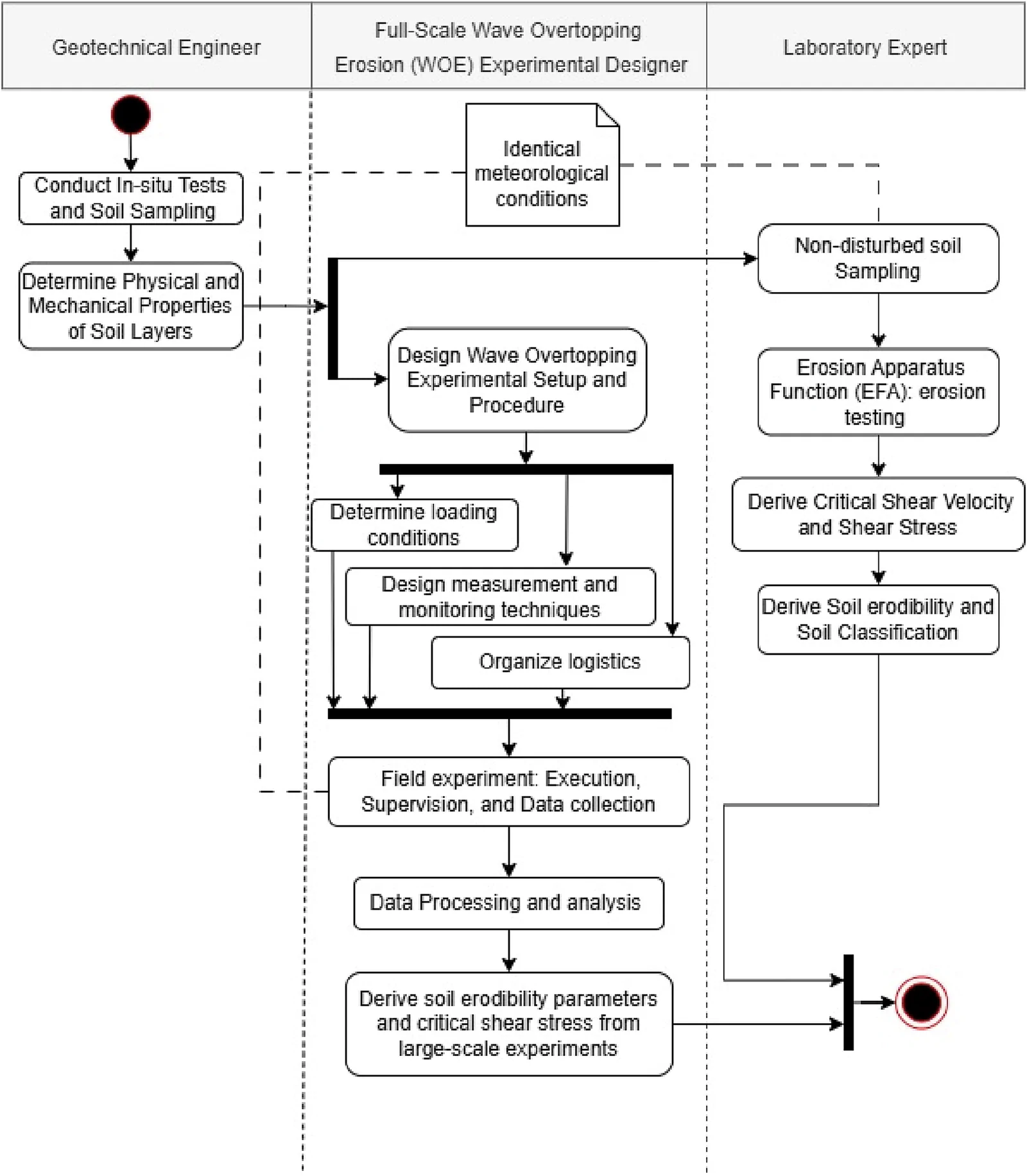
Unified Modeling Language(UML) activity diagram of wave overtopping experimental studies.

The overall experimental procedure and data production workflow are summarized in a Unified Modeling Language (UML) activity diagram (Fig. 4). This diagram illustrates the sequence of activities, their interdependencies, and the logical flow from site preparation through data acquisition, processing, and final dataset compilation.

The Methods section is organized as follows. First, the experimental campaigns are briefly described, highlighting the specific characteristics of each test case. This is followed by an overview of the geotechnical investigations performed prior to the experiments to determine representative soil parameters. The small-scale laboratory test, i.e., the Erosion Function Apparatus (EFA), used to independently assess the erosion resistance of the clay cover, is then described. Subsequently, the wave overtopping programs and the procedures used to determine the wavefront velocities are presented. The methods for recording and reconstructing the dike slope topography, including terrestrial laser scanning and Structure-from-Motion (SfM) photogrammetry, are then discussed, along with their application to the current dataset. Finally, a summary data table is provided, listing all parameters that should be predetermined or can be collected during or after wave overtopping erosion experiments.

### Field experiment campaigns

**Fig. 5:**
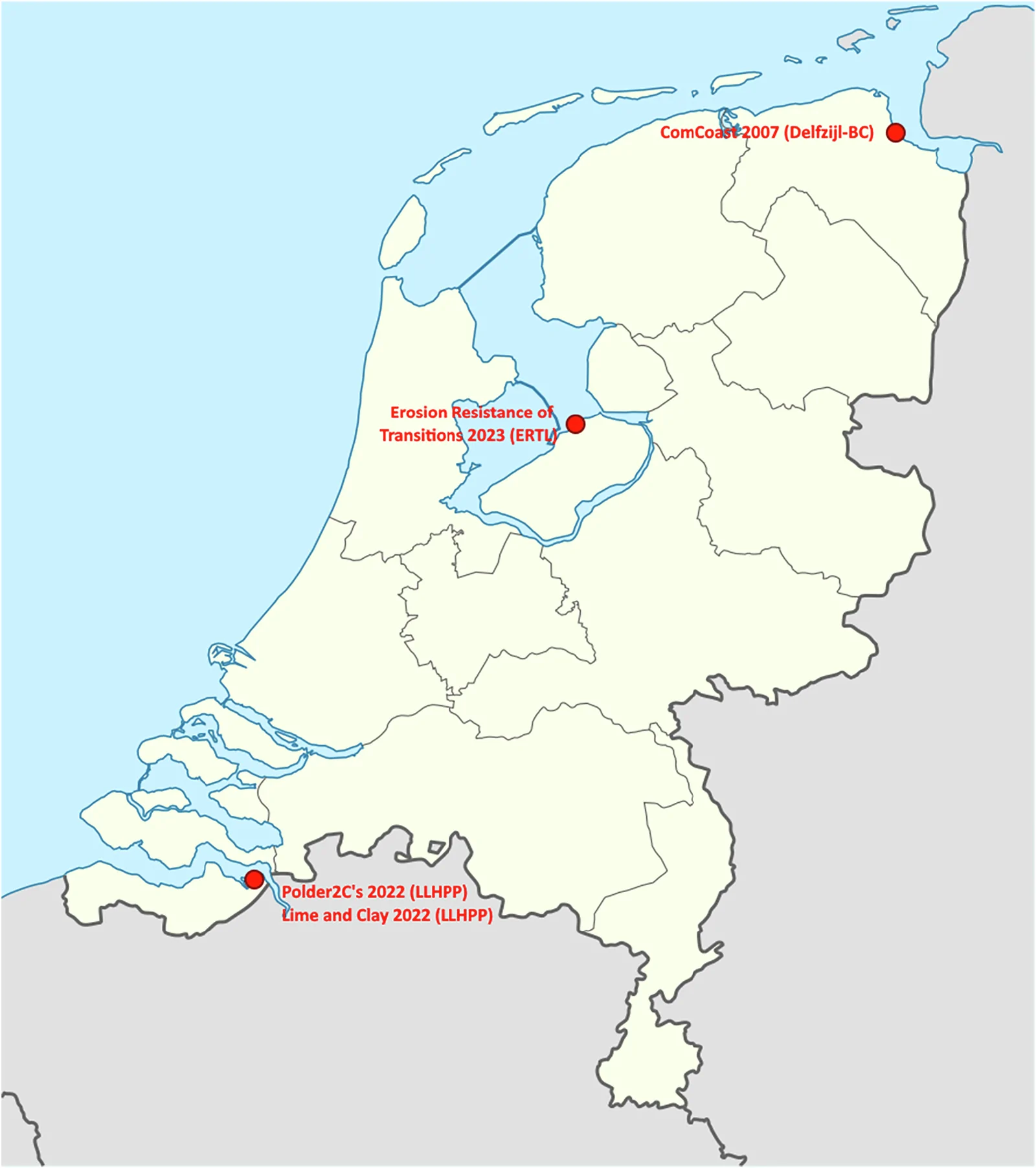
Geographical locations of the four experimental campaigns included in the database: ComCoast (2007, Delfzijl), Erosion Resistance of Transitions (2023, Lelystad/ERTL), Polder2C’s (2022, LLHPP), and Lime and Clay (2022, LLHPP). Base map adapted from Wikimedia Commons^30^ under CC BY-SA 3.0.

Figure 5 illustrates the locations of the experimental campaigns included in this database. The specific characteristics of each campaign are discussed in detail in the following sections.

### ComCoast project at Delfzijl

**Fig. 6:**
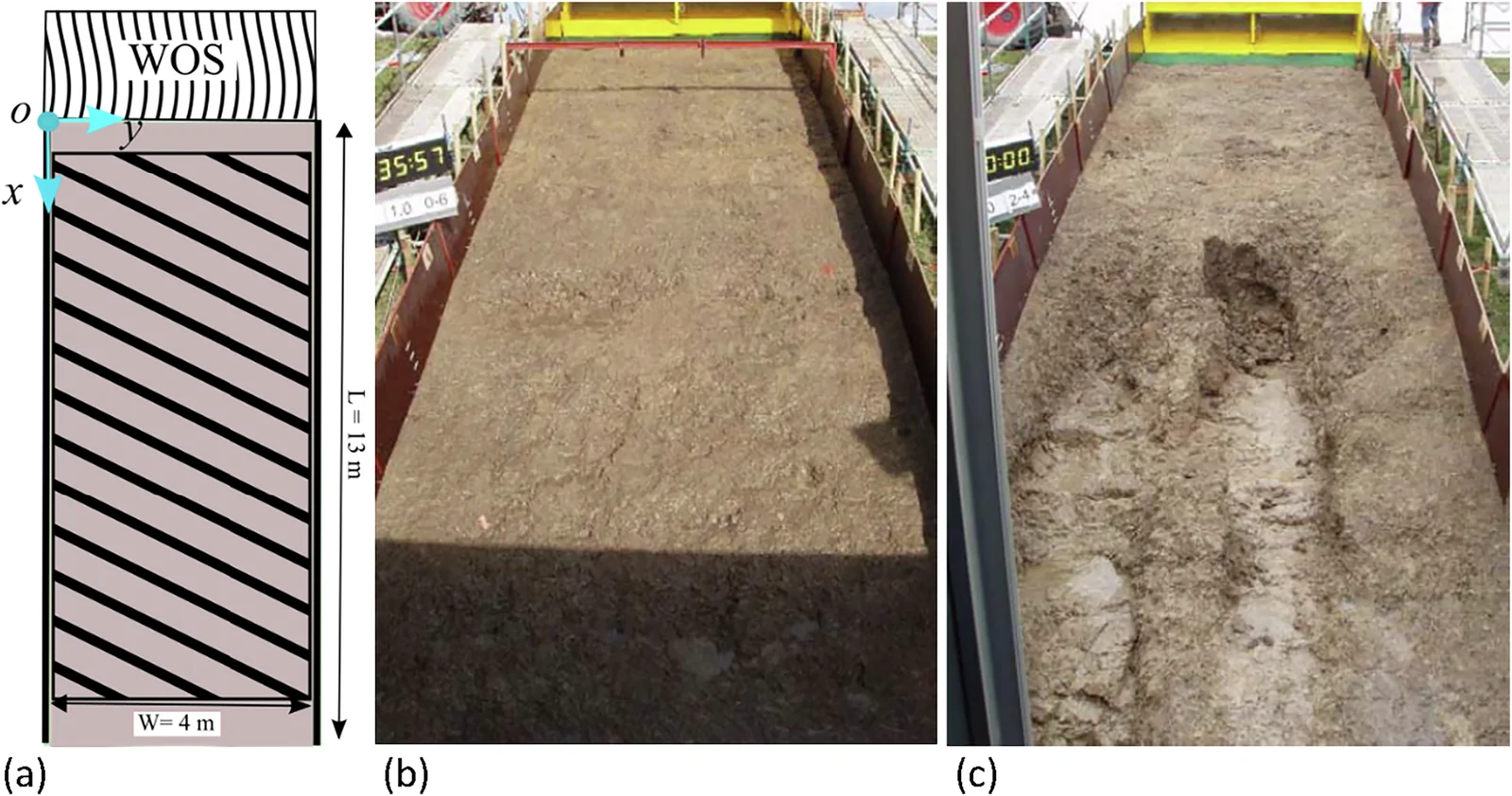
Delfzijl-BC experiment: (**a**) Layout and dimensions of test flume with a slope gradient of 1 V:2.8H, and photographs of the clay layer in (**b**) initial and (**c**) final states from ComCoast Report^10^.

The ComCoast project was dedicated to evaluating the *in-situ* erosion resistance of the landward slope of the sea dikes. For this purpose, a wave overtopping simulator^6^ had been designed to mimic the wave overtopping load on prototype dikes. Thereby, a series of experiments were conducted on a sea dike close to Delfzijl in the Netherlands with the aim to evaluate the performance of the grass-cover and the clay underneath when exposed to overtopping waves^10^. In order to test the erosion of the clay at one section the upper grass cover of 20 cm (sod) thickness was removed over the entire dike slope prior to the experiments Fig. 6a–c illustrates the experimental flume layout, alongside the initial and final soil surface states for the Delfzijl sea dike campaign. Still the traces of grass roots were present in the soil after the removal of top grass layer. Only the data from the bare clay test flume at Delfzijl is included in this paper and is referred to as Delfzijl-BC (Fig. 6).

### Polder2C’s project at LLHPP area

The INTERREG Polder2C’s project aimed at investigating current and innovative techniques, processes, methods and products in support of adapting the Interreg 2Seas Region to climate change. The experiments were conducted in the Living Lab Hedwige-Prosperpolder, which was in the process of being transformed into tidal nature area as part of a manager realignment project. The polder was located next to the river Schelde at the border between Belgium and the Netherlands. The old levee had to be demolished and was used to perform a wide range of experiments and practicing emergency responses.

**Fig. 7:**
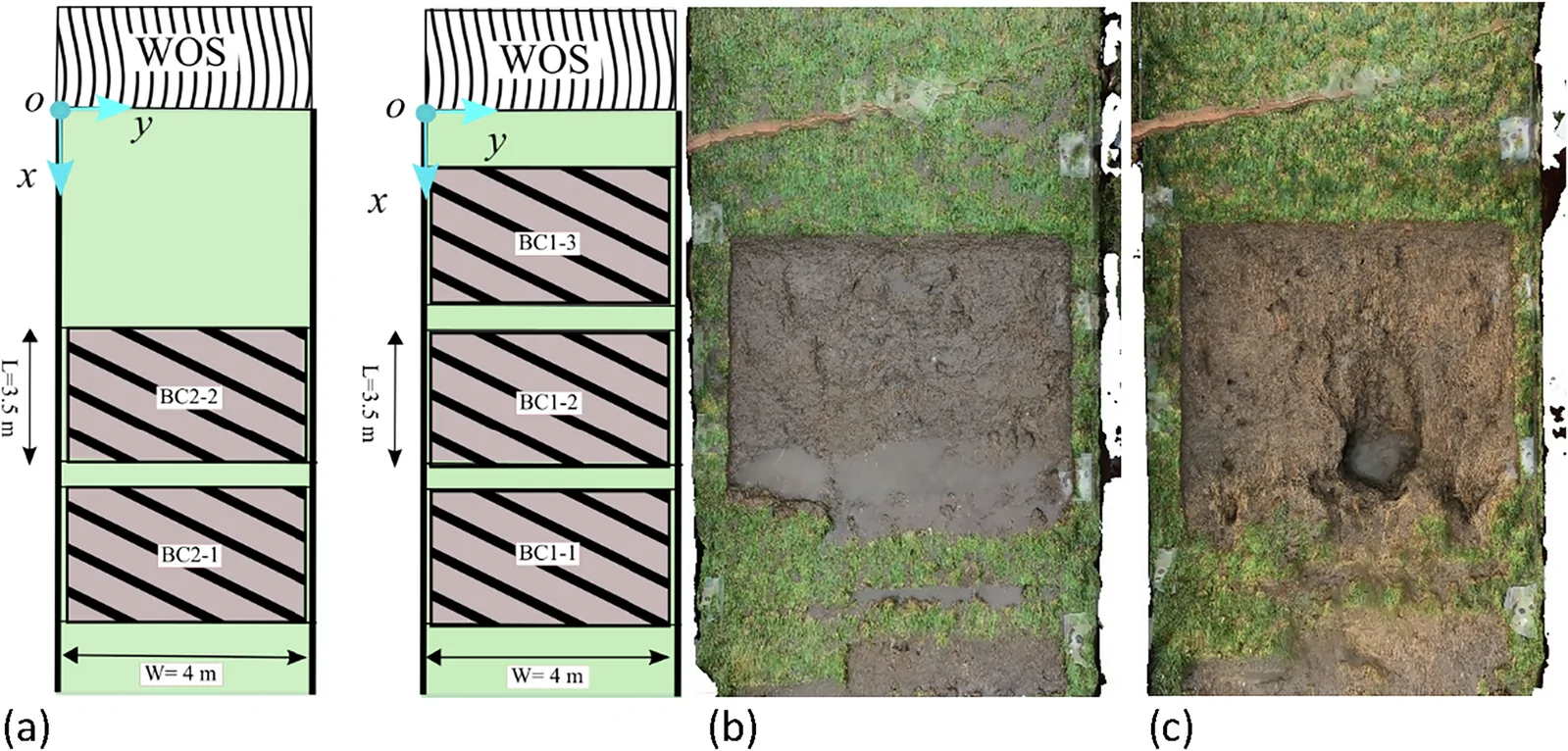
Overview of Polder2C’s experimental setup: (**a**) test layout including two flumes (BC1 and BC2); (**b**) detailed view of the initial clay surface; and (**c**) final state of the bare clay at section BC2-1 after the simulated storm program.

A series of experiments was aimed at assessing the residual strength of the clay underneath the grass cover. To study both the influence of the test location along the slope, and the magnitude and releasing order of waves, 2 test flumes and in total 5 test sections (see Fig. 7a) were constructed and tested^13^. In each test flume three test sections could be constructed along the landward slope of the dike. These zones were located respectively: close to the toe, at the middle of slope and close to the crest. The 20-cm upper grass cover was removed prior to experimentation over a length and width of approximately 3.5 m and 4 m, exposing 14 m² clay to the overtopping waves as shown in Fig. 7b. The test sections were prepared and tested from the toe towards the crest. As the flow velocities were super-critical, the impact of previous tests on lower located sections on tests executed at higher sections was negligible. This way multiple tests could be executed without the need of having to relocate the wave overtopping simulator. The first flume, BC1, was divided into 3 test zone i.e. BC1-1, BC1-2, and BC1-3 while the second flume constituted of only two tests sections, i.e. at the toe (BC2-1) and the middle of slope (BC2-2) as illustrated in Fig. 7a. The experiments halted as soon as the scour hole reached the sand core located at a depth of approximately 80 cm, to avoid rapid erosion of the flume (see Fig. 7c). At this point, the clay cover is considered to have failed in its function as erosion protection layer.

### Lime in Clay project at LLHPP area

**Fig. 8:**
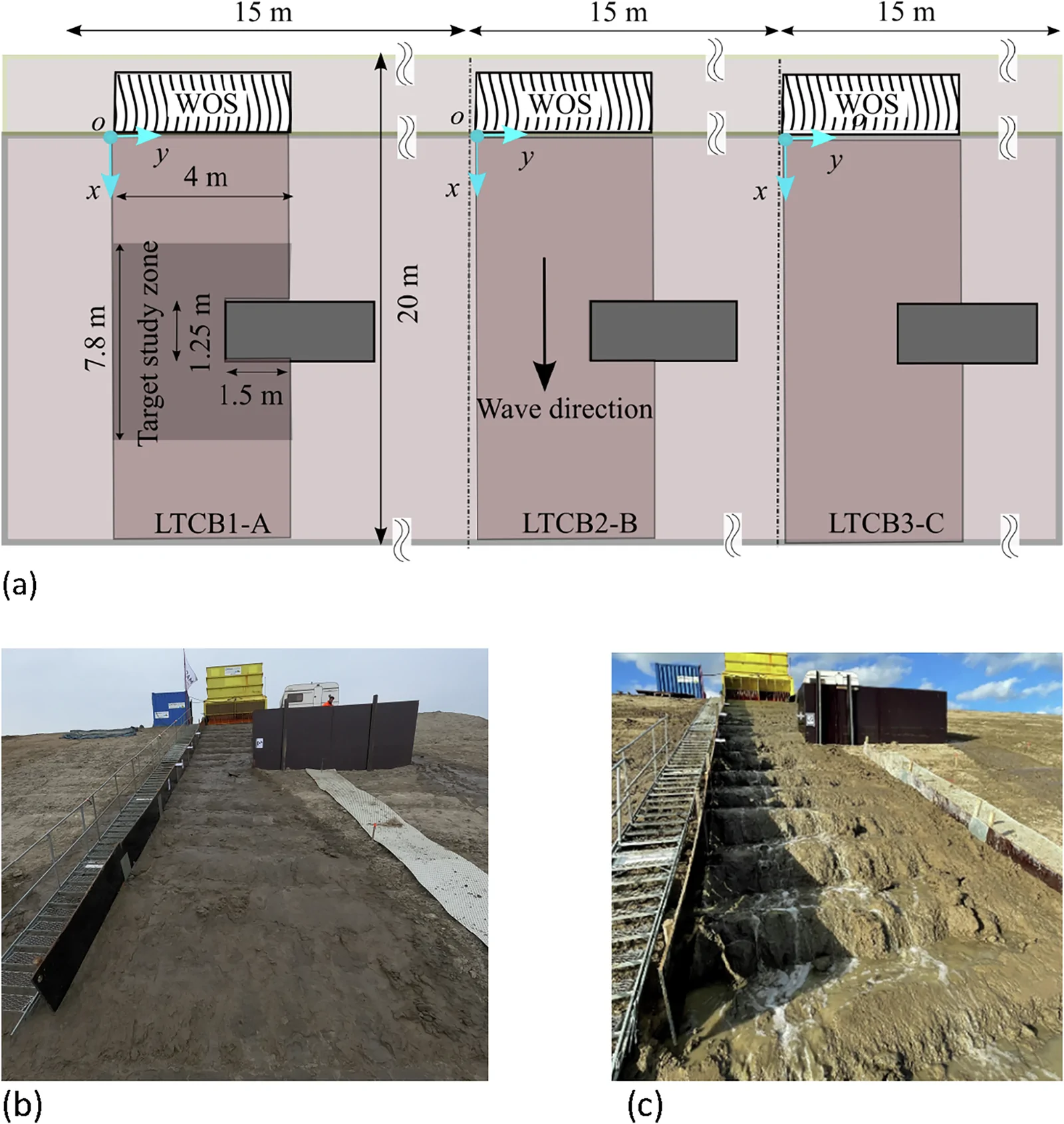
Lime in Clay Project: (**a**) test layout (**b**) initial state and (**c**) final state of test section LTCB3-C (Photographs by Infram).

A second series of experiments was performed within the Dutch Lime in Clay project on the same levee as the previous Polder2C’s campaign, though at different specific test sections within the LLHPP area. The objective of these series of tests was to investigate the erosion resistance of lime-treated clay at the soil-structure transition when exposed to wave overtopping loads (see Fig. 8a–c). In this context, the soil-structure transition is defined as the interface and the immediate area surrounding a solid physical obstacle placed within the clay layer, where local flow dynamics and soil erodibility are heavily influenced by the presence of the structure (indicated as target study zone in Fig. 8a). For that reason, the original clay levee cover was replaced with three newly constructed test sections (Fig. 8a), each featuring a distinct clay type stabilized with lime^13,21^. The characteristics of clay soils and percentage of lime added to the soil are provided in the geotechnical data. An example of such tests is shown in Fig. 8b,c.

### Erosion Resistance of Transitions project at Lelystad (ERTL)

A series of wave overtopping experiments was conducted on the main dike along Lake IJssel (IJsselmeer) near Lelystad, in the Netherlands. These experiments were carried out as part of a Dutch High Water Protection Programme (HWBP) innovation project aimed at investigating the effectiveness of reinforced transitions. These transitions were present between grass and soil, between different soil layers, and at the location of geometric transitions such as dike slope and road on the erosion resistance of the landward dike slope^14,22^.

**Fig. 9:**
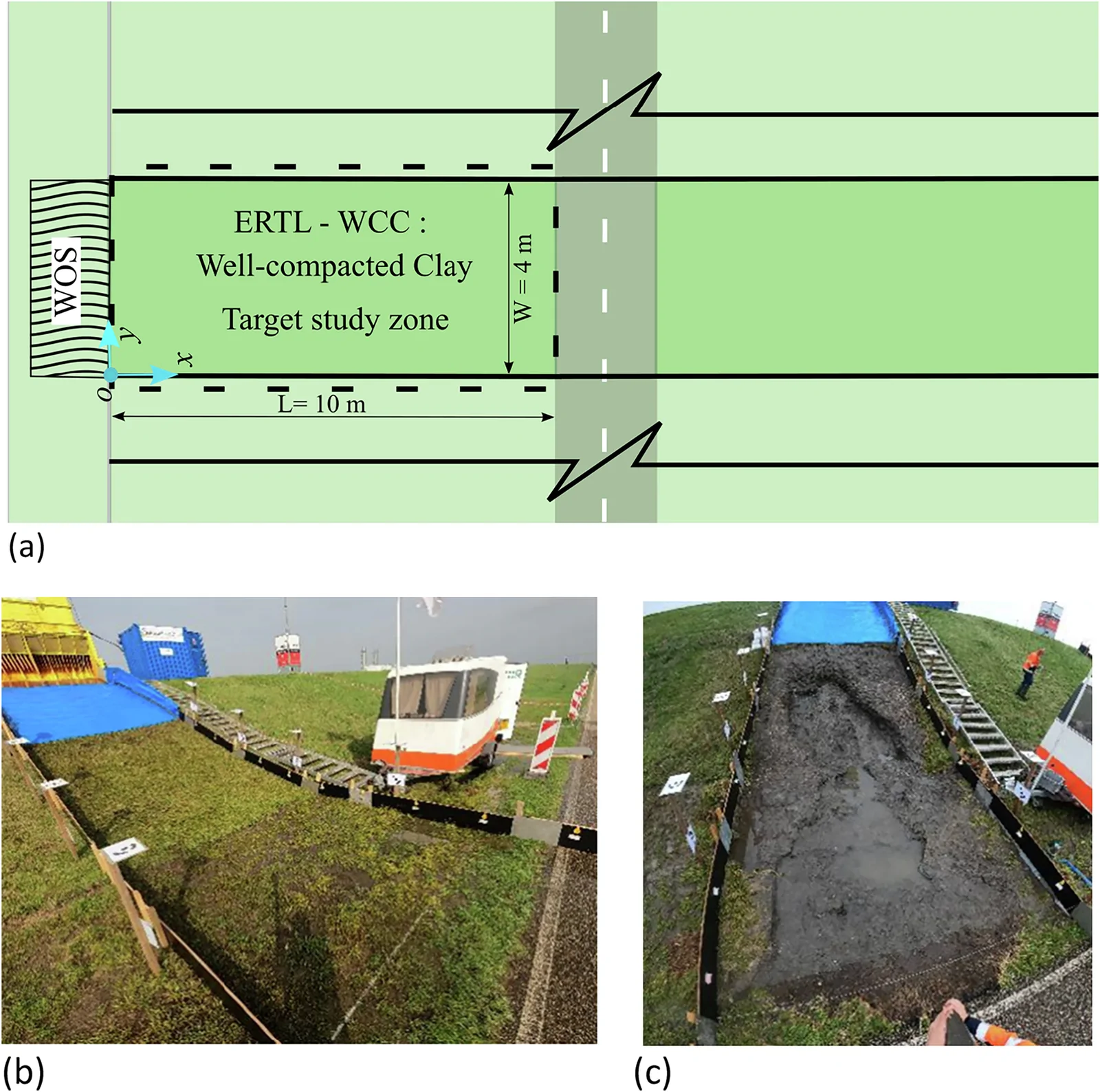
Wave-overtopping experiments at ERTL-WCC section (**a**) General test layout; (**b**) levee cover condition before the experimental program; and (**c**) state of the cover layer after the final test sequence (Photographs by Infram).

The database presented herein only covers the subset of the data collected during the experiment focusing on the erosion resistance of a newly constructed levee cover consisting of well-compacted clay reinforced with grass (Fig. 9a–c). This test was performed on the upper portion of the levee slope to study the transition between the slope and the asphalt road (Fig. 9a). The geometric transition was improved by adding 0.8 m of well-compacted clay, extending from the asphalt road up to a few meters along the upper slope. This layer theoretically replaces the original underlayer of natural clay.

This test is referred to hereafter as ERTL-WCC. It should be noted that the clay was placed only three months before the test, and grass turf was added approximately two months prior. These short establishment periods were insufficient for the grass root system to develop its full reinforcement capacity. The levee states before and after tests are shown in Fig. 9b,c.

### Geotechnical investigations of soil layers

Geotechnical surveys are a crucial preliminary step in experimental design, as they determine the physical properties and mechanical strength of the soil and grass cover before experiments begin. This information is essential for estimating the resistance of different levee cover layers, which are deterministic in the design of full-scale experiments being conducted.

For example, the following geotechnical investigations were conducted at the Delfzijl sea dike in support of the experimental work: (i) Soil Layer Composition and Grain Size Distribution were determined for the different layers (e.g., clay, sandy clay, sand);(ii) the shear resistance of the soil against sliding was determined through triaxial test; (iii) the water content was determined prior to the experiment as it is strongly correlated with shear strength; (iv) the grass root density was determined at different depths to assess the quality of the grass cover, which in turn provides insights into the structural integrity of the top layer; and finally, (v) the infiltration rate of water into the grass cover and soil was measured in order to understand the levee’s response to wave overtopping. A summary of these parameters is provided in Table 2, under the geotechnical data in the Data Record section.

### Erosion function apparatus (EFA) test

The Erosion Function Apparatus (EFA)^16^ was developed to evaluate soil erodibility and the relationship between erosion rate and water velocity or shear stress. Knowledge of soil erodibility is crucial for analysing problems related to soil erosion due to for example wave overtopping. The relationship between the erosion rate and shear stress at the soil-water interface is known as the erosion function^16,17,23^.

The apparatus itself consists of a rectangular channel with a cross-section of 101.6 mm × 50.8 mm and a length of 1.22 m, through which pressurized water flows under controlled conditions. To ensure saturated conditions, the test channel is inundated with water for one hour prior to testing. This equilibration period allows for the complete saturation of the soil sample before the application of the first flow velocity increment.

**Fig. 10:**
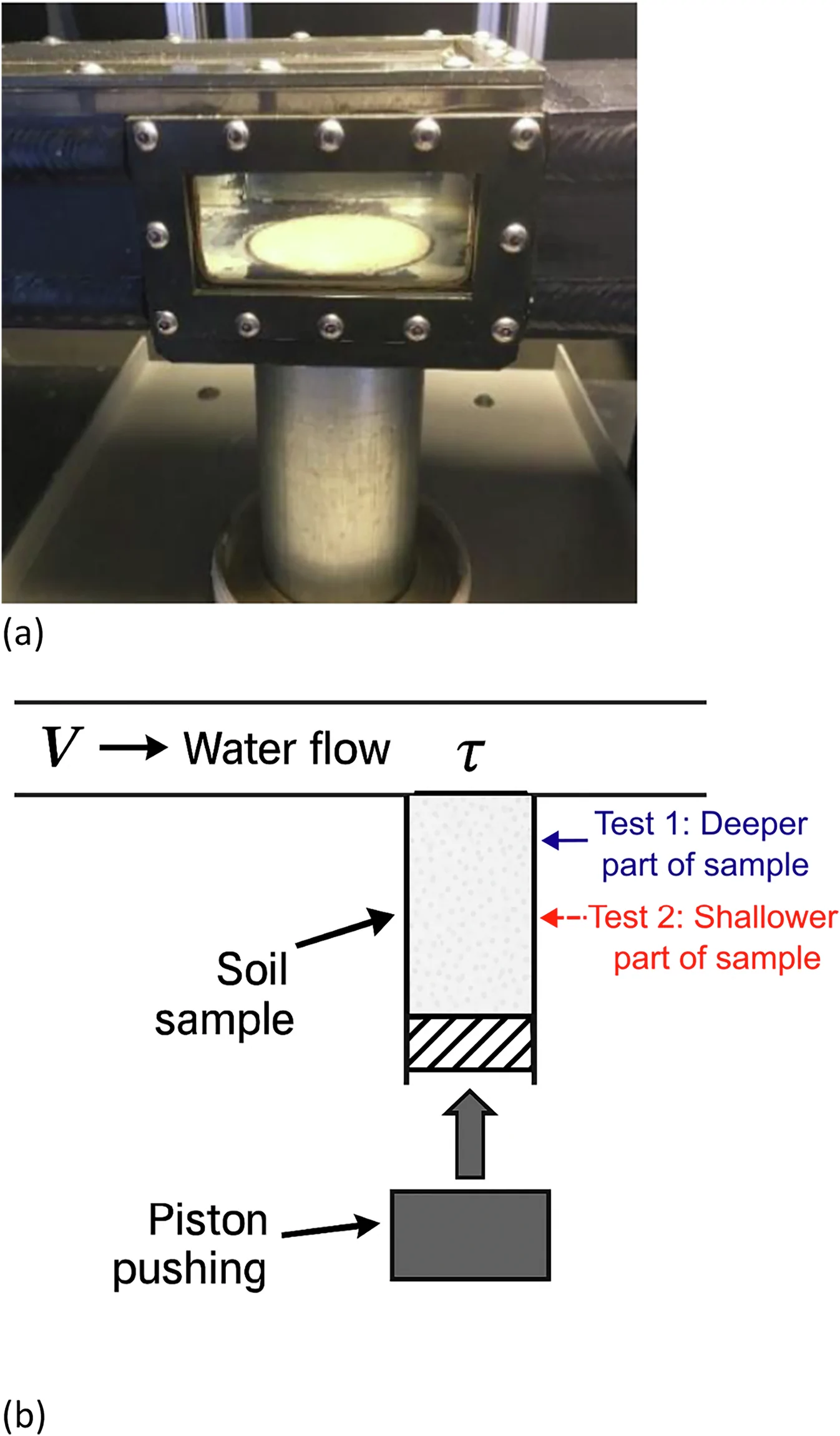
The EFA apparatus, showing (**a**) the position of the Shelby tube containing the soil sample, and (**b**) a schematic representation of the water flow.

EFA tests can be conducted on soil collected directly from the field or on soil prepared in the laboratory. In both cases, a Shelby tube (76.2 mm diameter, ASTM-D1587) is used, either for coring or for containing the laboratory-prepared soil. In the laboratory, testing begins by placing the Shelby tube with the soil into the EFA apparatus (see Fig. 10a). One end of the Shelby tube rests on a piston, while the other extends through a circular opening at the bottom of the channel. The top of the sample is placed flush with the base of the flow pipe, ensuring a smooth flow over the soil. At the transition between the channel and the sample a difference in bed roughness may be present. The channel is filled with water. After one hour the test is started whereby a flow is generated over the sample. A piston at the bottom end of the Shelby tube pushes the soil sample upward at the same rate as it is eroded by the flowing water (see Fig. 10b). In practice, this synchronization is achieved through continuous manual adjustment by the operator, who pushes the sample upward in 0.5 mm increments to ensure the soil surface always remains flush with the bottom of the channel. Because the piston is adjusted to the erosion rate, its advancement directly measures the eroded thickness. The advancement of this piston equals the erosion rate. For each soil sample, two successive tests can be performed following the EFA protocol, each consisting of several imposed flow velocities. The first test (Test 1) begins by eroding the deeper portion of the sample, as the Shelby tube is inverted before placement in the device. After this portion is fully eroded, the setup continues to Test 2, corresponding to the shallower part of the same sample as shown in Fig. 10b. The test is carried out in speed increments. For each speed, there are two stopping conditions for measuring erosion: erosion measurement is stopped after 30 minutes. If 50 mm of soil is eroded before these 30 minutes of continuous flow (particularly for slightly erodible soils), or if 50 mm of soil is eroded before these 30 minutes are up (particularly for highly erodible soils). Before moving from one flow velocity step to the next, the sample is leveled again to correct any irregular erosion pit geometries.

A flowmeter, placed in line with the water flow, provides the water velocity from which the interface shear stress is calculated using Moody’s chart. The flowmeter has an absolute uncertainty of 0.09 m/s (1% of the 9 m/s full scale). Because this absolute uncertainty remains constant, the relative uncertainty increases inversely proportional to the measured velocity (e.g., 2% at 4.5 m/s, but 18% at 0.5 m/s), making flow velocity uncertainty the dominant factor in determining shear stress uncertainty.

**Fig. 11:**
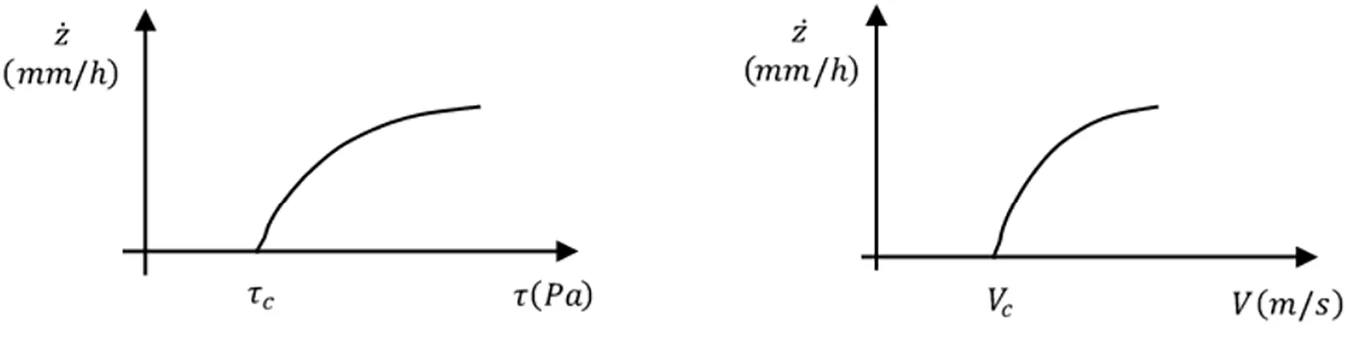
Erosion function relationships defined for EFA tests adapted form Briaud *et al*.^23^.

Erosion function relationships, such as those shown in Fig. 11 adapted from Briaud *et al*.^23^, illustrate the produced erosion curves. These can be plotted as a function of velocity or shear stress ((ż, V) and (ż, τ)) although shear stress (τ) is the most used and theoretically preferred parameter in erosion modelling. These graphs are generated from EFA test results using USCS soil classification^23^. They are intended to provide preliminary information on soil erosion behaviour, offering a first-order estimate of soil erodibility. The primary purpose is to reduce the need for extensive systematic erosion testing if these initial estimates indicate that the soil is not prone to problematic erosion.

### Wave overtopping programs

The Wave Overtopping Simulator (WOS) shown in Fig. 1c was developed to replicate overtopping events due to normal incoming waves with a wave steepness of 4%. The simulator operates by storing and releasing specific volumes of water at predetermined times, mimicking the “tongue” of a real overtopping wave^6^. The characteristics of the incident waves, such as significant wave height (*H*_*s*_), average and peak periods (*T*_*m*_, *T*_*p*_) and wave steepness (*s*_*ov*_), determine the volumes (*V*) and frequencies of overtopped waves (*P*_*V*_). These parameters are used in prediction models, such as those in the EurOtop manual^24^, to calculate the probability of overtopping (*P*_*ov*_) and the 2%-wave run-up level (*R*_*u2%*_), which, in turn, inform the design of the simulated overtopping events. The characteristics of incident waves and geometrical parameters used to generate the overtopping events are detailed in Table 3, located within the data record section.

The distribution of wave volumes released from the Wave Overtopping Simulator (WOS) follow a modified Weibull distribution given in Eq. (3). The original distribution has a shape factor of 0.75 and a scale factor, *a*, given by Eq. (4). The distribution is modified in order to fall within the maximum volume of the simulator.

3 $${P}_{V}=P(\tilde{V}\le V)=1-{e}^{-{\left(\frac{V}{a}\right)}^{0.75}}$$

4 $$a=0.84{T}_{m}\frac{q}{{P}_{ov}}$$

Where *q* is the mean overtopping discharge per unit width.

**Fig. 12:**
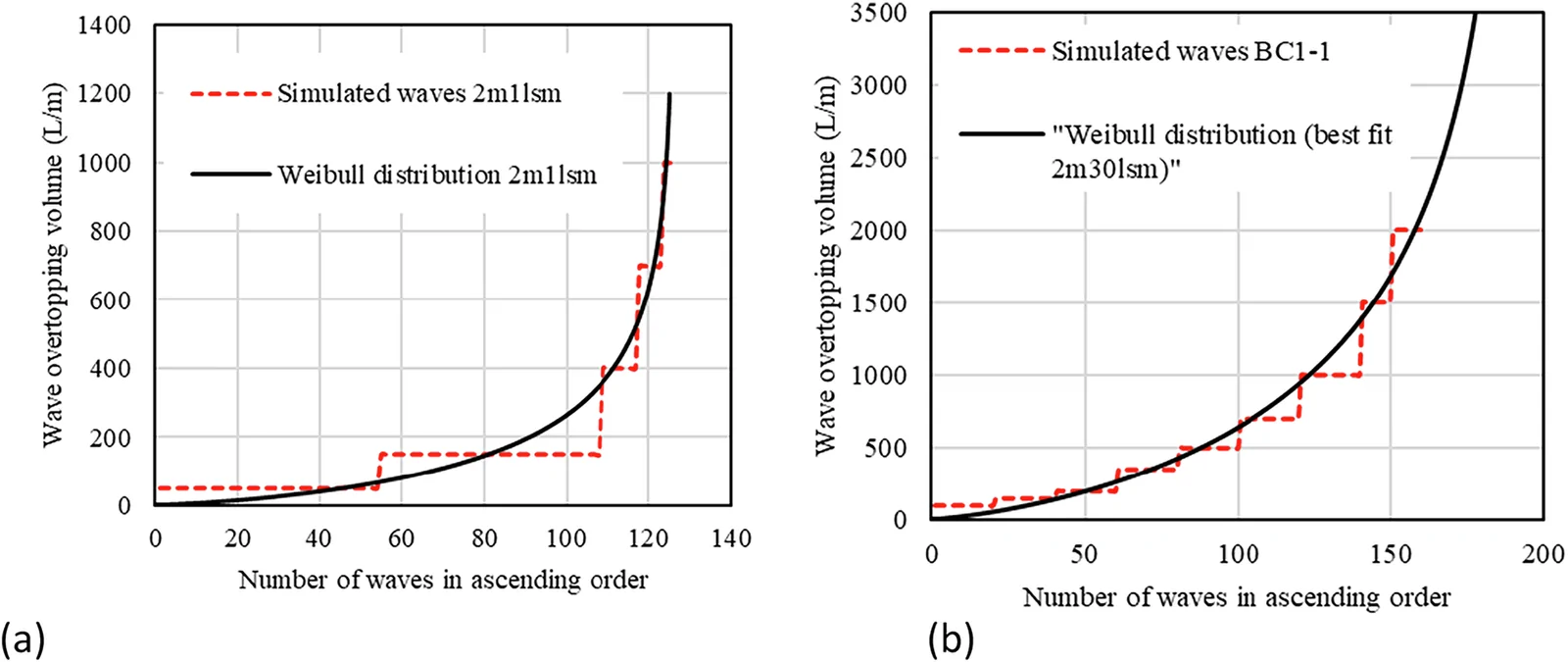
Weibull wave distribution vs simulated wave distribution by WOS at (**a**) Delfzijl BC-2m1lsm, and (**b**) LLHPP-BC1-1.

To illustrate this, the results of Weibull distribution and the waves simulated by the WOS for two specific wave programs are shown in Fig. 12a,b. One program, 2m1lms (representing H_s_ = 2 m and q = 1 l/s/m), corresponds to the Delfzijl-BC test (Fig. 12a), while the other, 2m30lms (H_s_ = 2 m and q = 30 l/s/m) corresponds to the LLHPP -BC1-1 test (Fig. 12b).

**Fig. 13:**
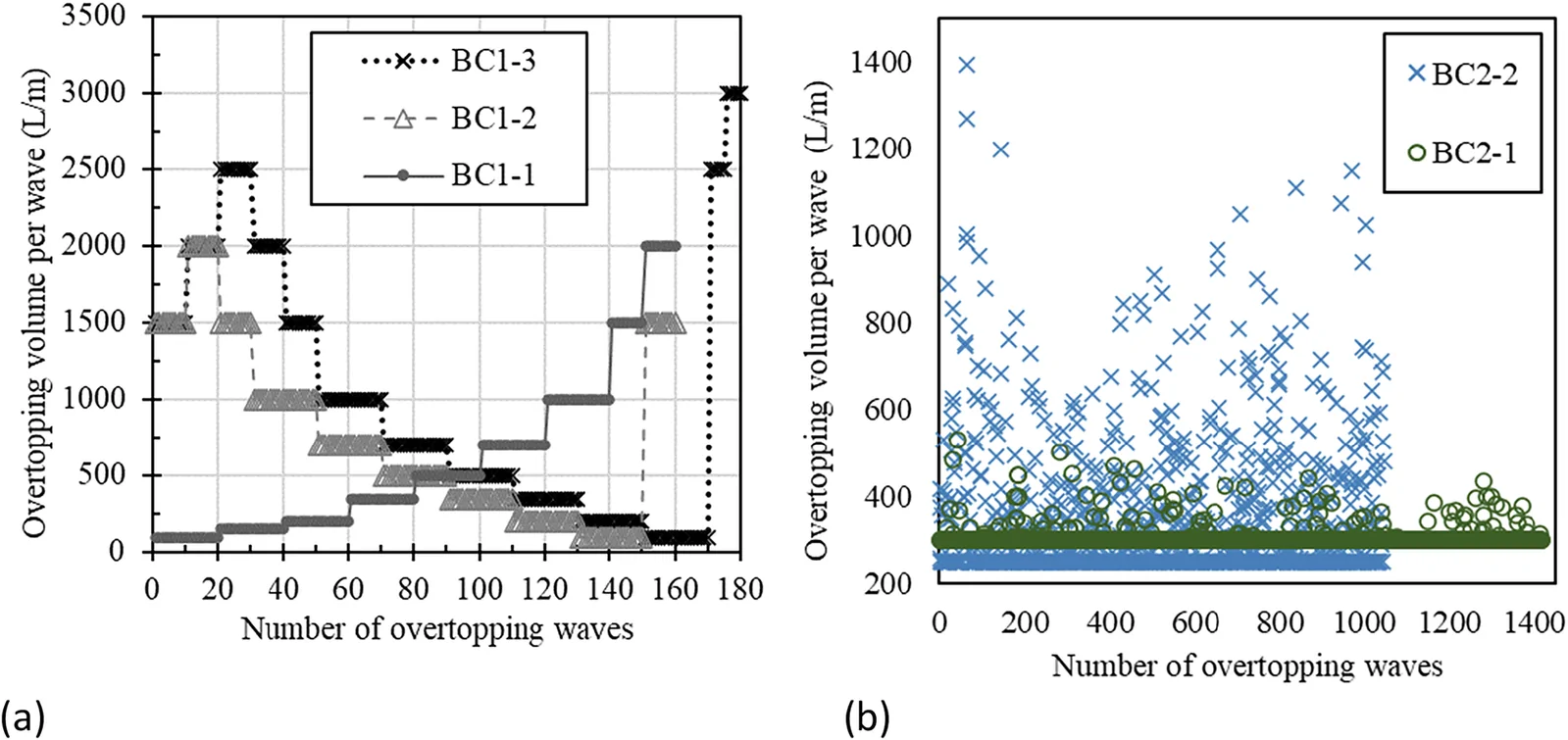
Overtopping waves realised in (**a**) specific orders (regular) and (**b**) random order (irregular) simulated in BC1 and BC2 experiments at LLHPP.

In all cases, these waves were released randomly to mimic a real storm causing normal incoming waves to overtop the levee. The only exceptions were the LLHPP-BC1 tests, where waves were released in specific, deliberate patterns to study the effect of the order and magnitude of the waves on erosion. Figure 13a,b illustrate these patterns in more detail, showing the delibrate or (“regular”) wave patterns for LLHPP-BC1 tests and the random (or “irregular”) waves patterns for LLHPP-BC2 tests.

The overall wave overtopping programs executed across all the studies in this dataset are presented in Figs. 19, 20 located in Data record section.

### Wavefront velocity

**Fig. 14:**
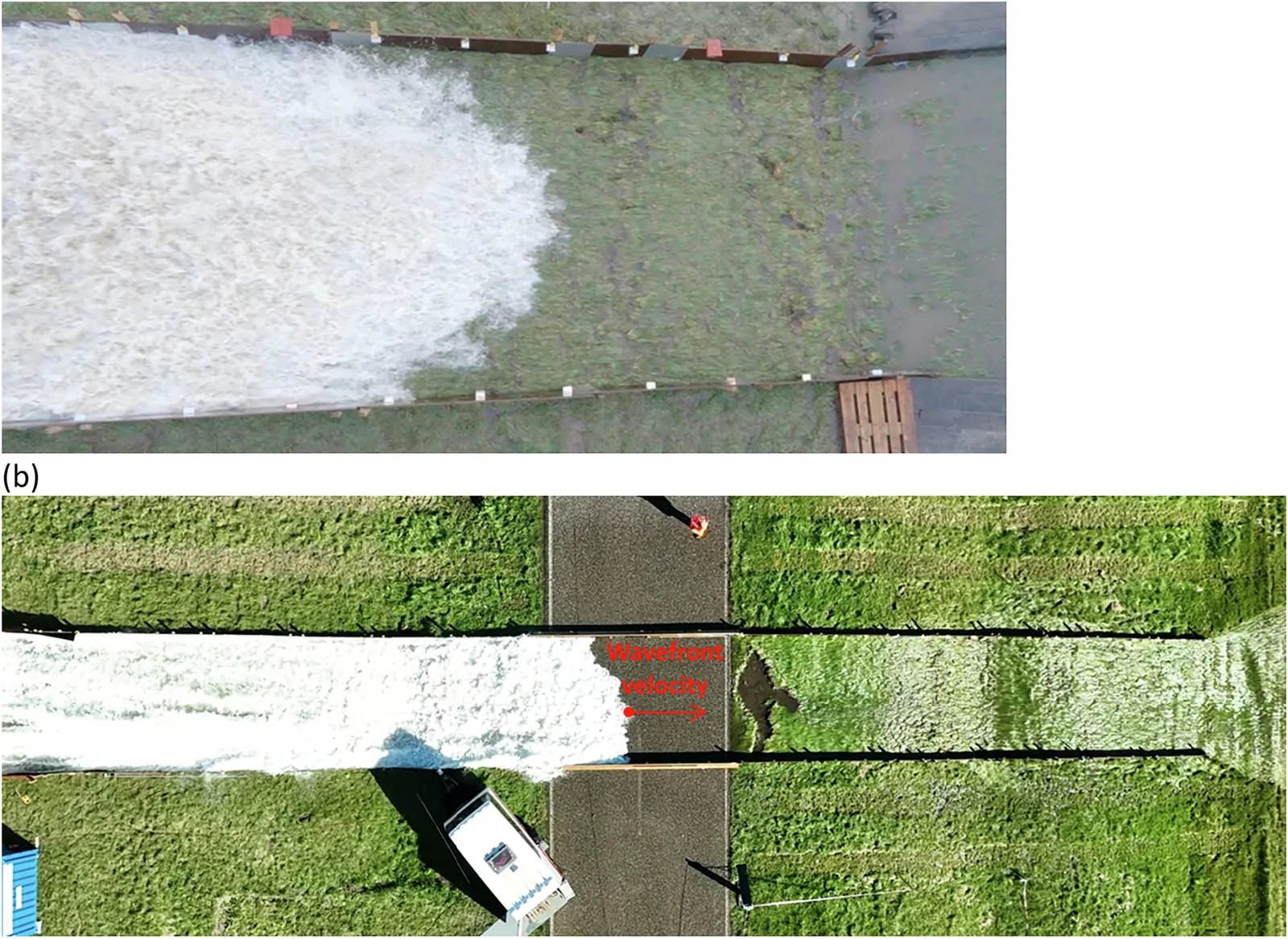
Wavefront velocity is determined by tracking the front of an overtopping wave: (**a**) Polder2C’s project, and (**b**) ERTL field experiments^27^ Video footages by Infram (wavefront indicated by the red point and arrow).

An important parameter related to the hydrodynamics of an overtopping wave is the wavefront velocity of an overtopping wave (see Fig. 14a,b). The peak stresses exerted by the overtopping waves are approximately proportional to the square of the front velocity^25^. As a result, models such as the cumulative overload method, make use of this wavefront velocity to quantify the hydraulic loading by an overtopping wave^7,26^.

The wavefront velocity can be determined from video footage taken during field experiments. Physical reference marks were installed at one-meter intervals along the side walls of the test flume (visible in Fig. 14a). During manual post-processing, these physical markers served as visual anchors to define transversal lines across the test section. The wavefront velocity is estimated by counting the frames needed for the wavefront to travel one meter. This analysis is typically performed on the video footage from the hydraulic measurements, where the wave overtopping simulator releases increasing volume from 250 to 5,000 l/m. This results in a dataset of the wavefront velocity along the tested section for each released volume. In the post, two methods have been used to obtain these wavefront velocities:

1. Manual analysis (frame-by-frame)
2. Tracking the wavefront using video detection (Wavefront Tracker)

The manual analysis has been widely used in recent field experiments to determine wavefront velocity. In this approach, video footage is examined frame by frame to identify the exact moments when the wavefront passes each meter mark along the slope. However, this method is time-consuming and labor-intensive.

**Fig. 15:**
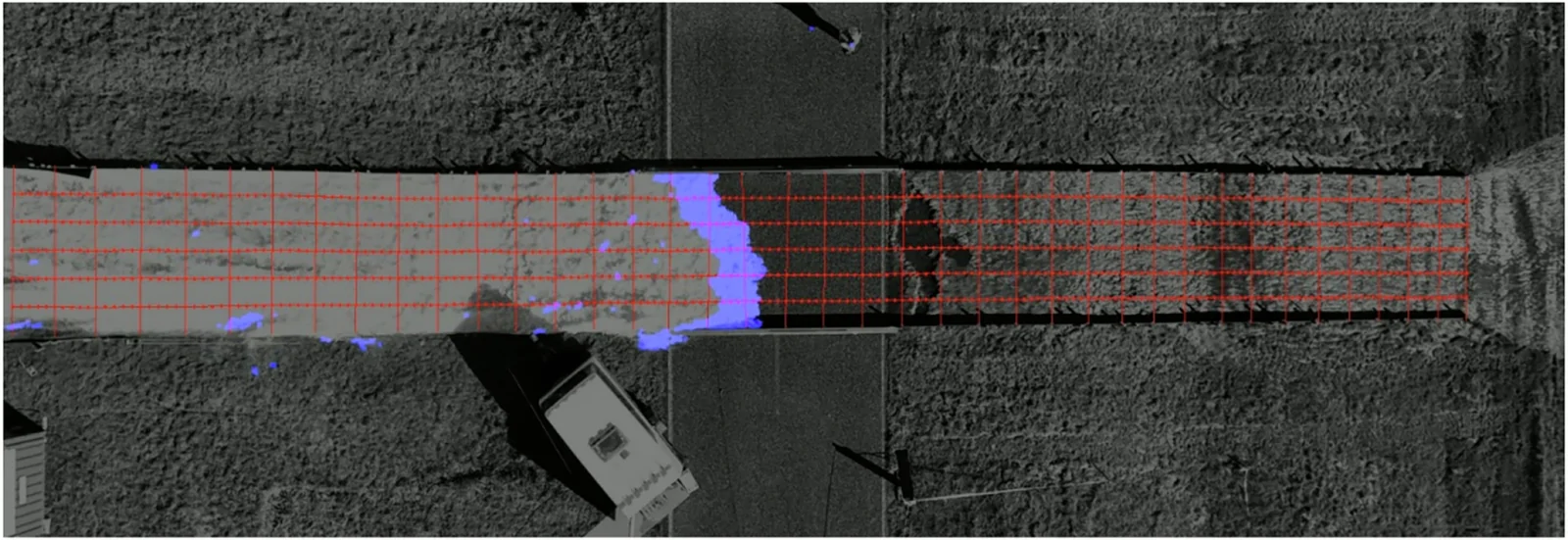
The Wavefront Tracker determines the space-time relationship of the foaming white of the wavefront (highlighted in blue). Video footage of ERTL field experiments by Infram.

The Wavefront Tracker is a video detection tool in Python to automate this process and is developed for the ERTL experiments at IJsselmeerdijk^27^. After indicating the meter marks, the Python model will automatically track the foaming white wavefront and calculate the wavefront velocities (see Fig. 15). One of the benefits of this model is that the spatial step of the grid can be made smaller at high resolutions, yielding more data points.

### Morphological and erosion monitoring —SfM-Photogrammetry

To monitor and measure erosion progression in the levee cover layer during overtopping experiments, close-range Structure-from-Motion^13,28,29^ (SfM) photogrammetry was implemented. SfM is a cost-effective and versatile technique for acquiring high-resolution topographic data and has been successfully applied to a wide range of complex geometrical conditions and scales^29^. Its accuracy has been validated in numerous studies through direct comparison with terrestrial laser scanning^29^ and other GIS-based methods^13^.

**Fig. 16:**
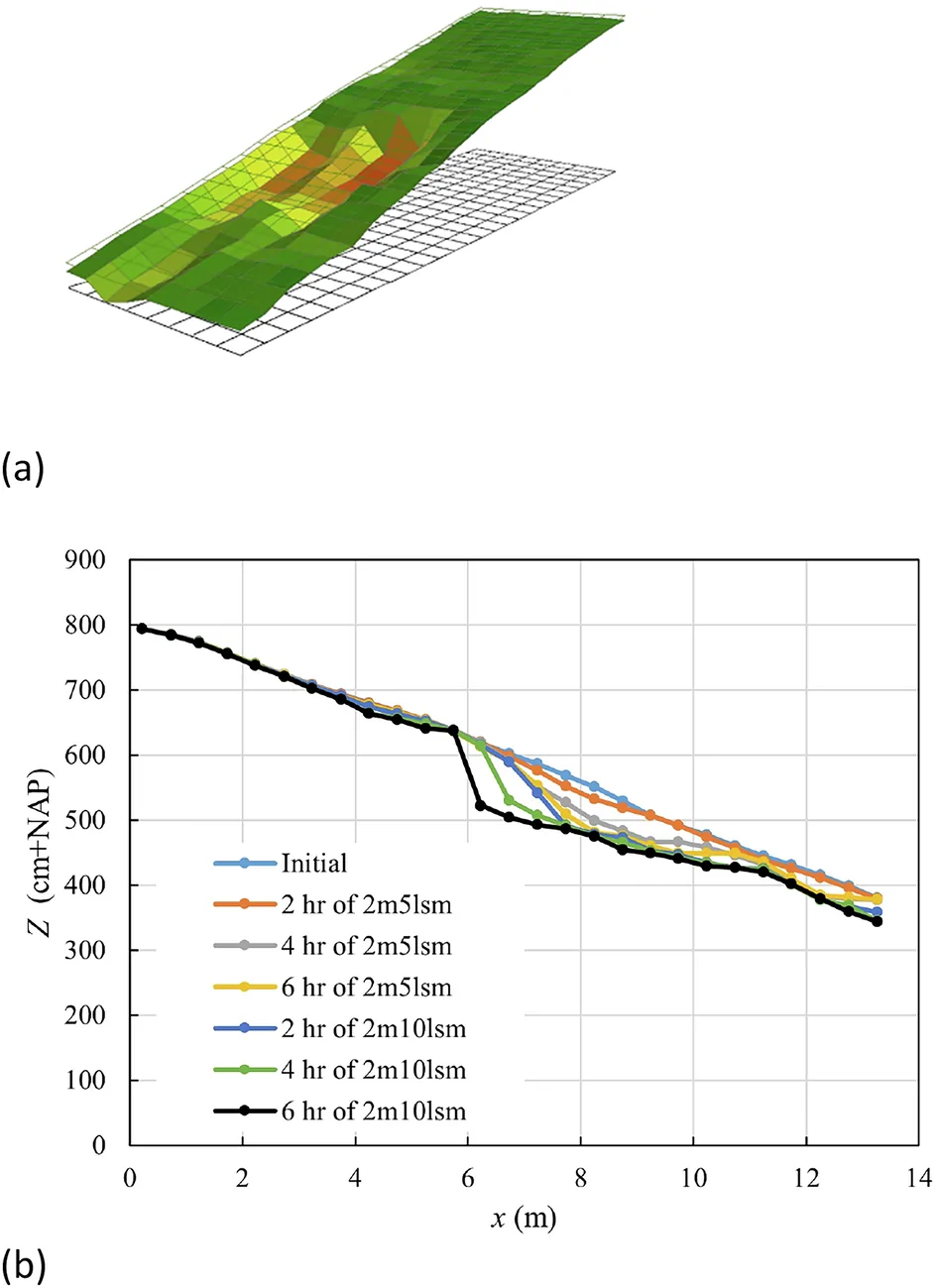
Dike slope topography measured (**a**) manually, and (**b**) derived dike slope profiles showing the morphological evolution during the Delfzijl-BC wave overtopping tests (profiles correspond to the transversal section where maximum bed level changes occurred across the test flume).

**Fig. 17:**
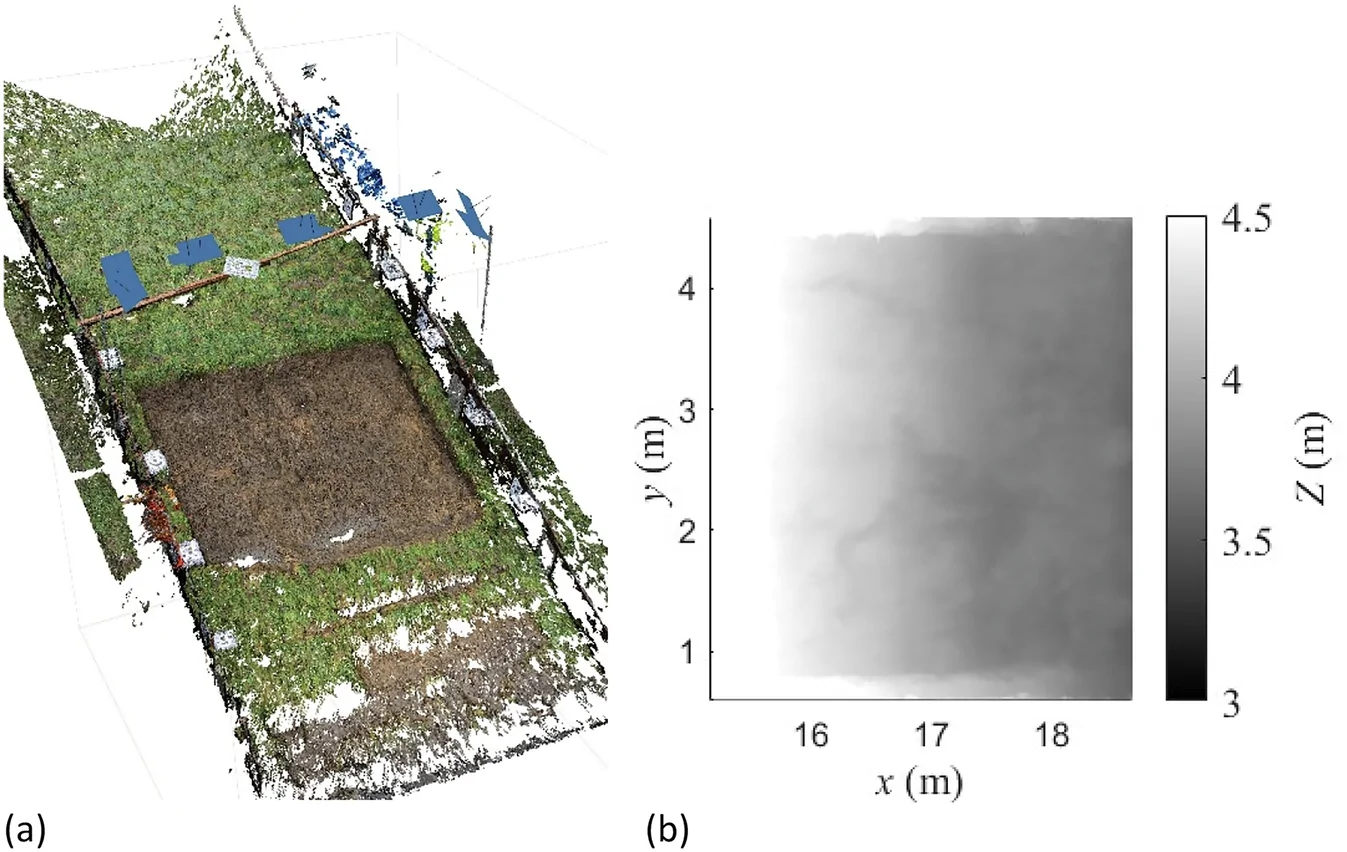
Application of SfM-Photogrammetry using a multiple fixed-camera system: (**a**) dense point cloud and camera layout (blue rectangles indicate the fixed camera positions and orientations), and (**b**) resulting DEM.

**Fig. 18:**
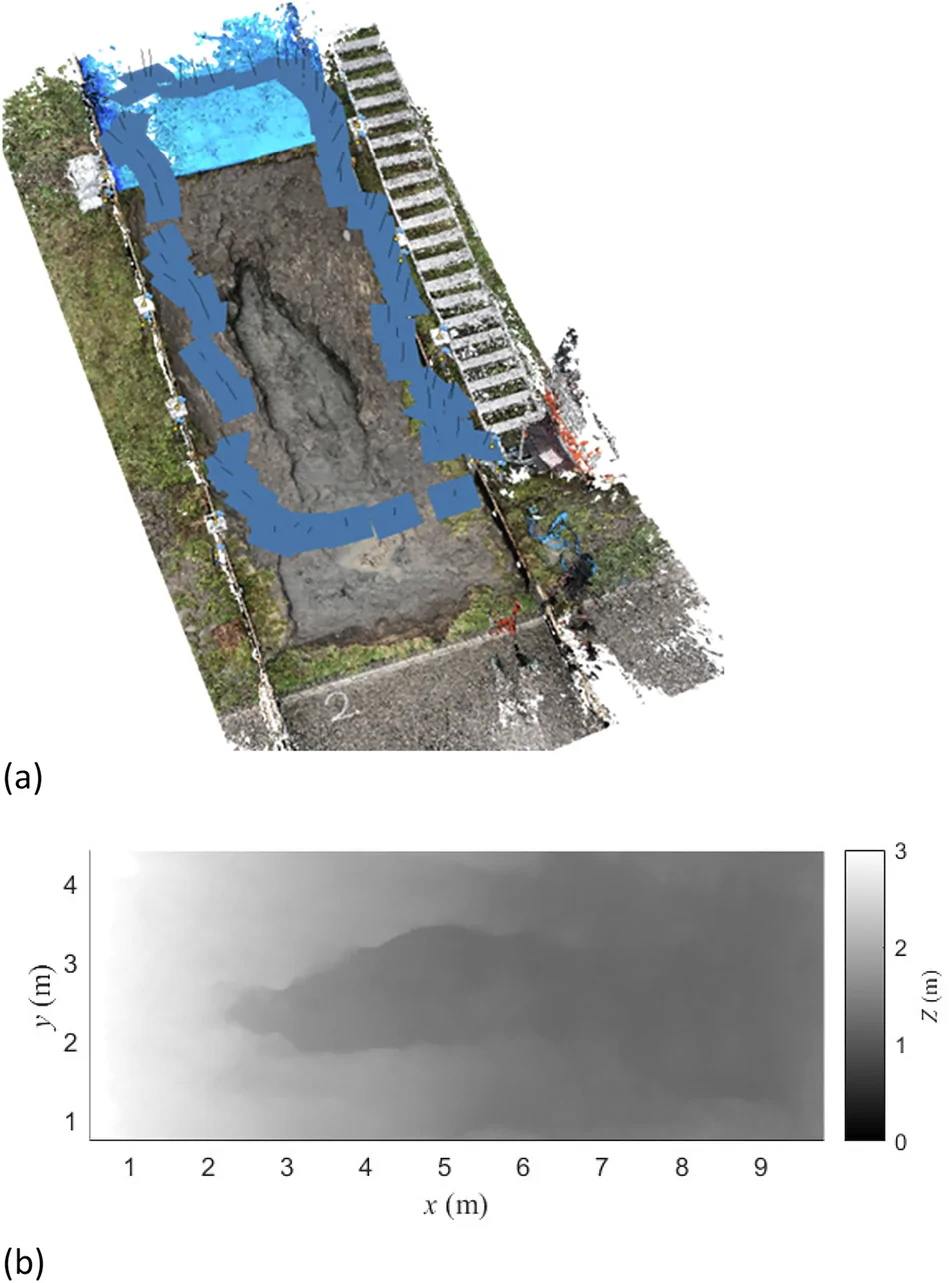
Application of SfM-Photogrammetry using a mobile camera: (**a**) dense point cloud and camera layout (blue rectangles indicate the mobile camera positions and orientations), and (**b**) resulting DEM.

In this study, 2D and 3D dike surface models reconstructed from manual pointwise measurements (Fig. 16a,b) and SfM dataset (Fig. 17a,b) were used to precisely track morphological changes over time. These models, shown in Figs. 16b, 17b, 18b, provide the basis for quantifying erosion rates and assessing soil erodibility.

SfM-Photogrammetry processing was performed using Metashape Agisoft software (see Code Availability section). The technique was applied in two distinct photo acquisition configurations^13^: (i) Multiple Fixed Camera System: Ten fixed GoPro Hero 9 cameras (San Mateo, California) mounted on a rigid structure enabled continuous photography of the test zone (see Fig. 17); (ii) Mobile camera system: A mobile camera mounted on a monopod was carried around the test zone to capture photos (see Fig. 18). In both configurations cameras were set to time-lapse mode (one photo per second) using wide-angle (Fisheye) lenses. The dense point cloud models reconstructed from these photos, shown in Figs. 17a, 18a, resulted in a high resolution of 1.25 mm/pix. In order to produce Digital Elevation Models (DEMs) in real-world coordinates, ground control points (GCPs) were placed. These fixed, identifiable reference points were distributed both horizontally and vertically around the test flumes. Their coordinates were recorded using an RTK GNSS GPS tool in the Amersfoort / RD New coordinate system (EPSG:28992), where the X and Y directions represent Easting andNorthing in this coordinate system (Fig. 3). The Z-direction, or elevation, is given relative to the Normaal Amsterdams Peil (NAP, Amsterdam Ordnance Datum). A brief description of the photogrammetry workflow and its precision is provided in the Technical Validation section.

## Data Record

The data record associated with this work^5^ is stored in the Open Data UCLouvain Dataverse repository, which is a public access repository for publishing research data.

This dataset consists of 4 zipped folders corresponding to 4 experimental campaigns i.e. ComCoast_Delfzijl, Polder2Cs_LLHPP, Lime_Clay_LLHPP, and ERTL_Lelystad.

Each experimental campaign folder contains several subfolders on (i) geotechnical investigations, (ii) reduced size experiments containing EFA test results, and (iii) Full scale WOE Experiments. The Full-scale WOE Experiments folder contains separate subfolders for each test section that include the data from field experiments. The field data is divided in two categories i.e. detailed wave overtopping conditions and programs as well as photogrammetry models to record the morphological evolution of levee slope during the experiment. As an example, Table 1 illustrates in detail each subfolder and containing data record for Polder2Cs_LLHPP and test section BC1-1.

## Data Overview

### Geotechnical data

The dataset includes results from geotechnical investigations provided both as original reports and as detailed summary tables in CSV format within the respective test campaign folders in the data repository (e.g., *Geotechnical_Inves_Data_ComCoast_Delfzijl.csv* and *Geotechnical_Data_Polder2Cs_LLHPP.csv*). A global summary of the key parameters measured across the different campaigns is presented in Table 2. The “Acronym” column in Table 2 serves as a reference key, defining the exact variable identifiers used within the corresponding CSV files.

**Table 2:** Summary of soil parameters and geotechnical tests.

Test and Parameter Definition	Acronym	Unit
Grain size distribution	Granulometry curves	
Characteristic diameters	*d*_50_	mm
Water (moisture) content	_*w*_	(%)
Dry unit weight	γ_dry_	(kN/m³)
Atterberg limits *i.e*., Liquid Limit, Plastic Limit, Shrinkage Limit, and derived parameters *i.e*., Plasticity Index and Liquidity Index	LL, PL, SL, PI, LI	(%)
Consistency index	*Ic*	(−)
Sand content, Lutum content, Silt content, Salt content, Organic matter		(%)
Consolidation test parameters *i.e*., Compression index, Recompression index, Preconsolidation Pressure, Coefficient of consolidation	Cc, Cr, Pc, Cv	(−), (kPa), (m²/s)
Triaxial or direct shear stress tests derived parameters *i.e*., cohesion and internal friction angle and	_C, φ_	(kPa), (°)
Collapse test		(%)
Erodibility coefficient and critical shear stress obtained from *in-situ* Jet Erosion Test (JET), and Fire Hose Erosion Test (FHET)	k_d_, τ_c_	(cm³/(N.s), (Pa)
Grass root density, pull out test (the anchorage capacity and tensile strength)	(−), F_max_, T_s_	(Score 1–5), (N), (kPa)
Hydraulic conductivity and infiltration rate	K, k	(m/s), (m/day)

Whenever these parameters are available, a range of representative values is given directly in the campaign-specific CSV summary tables. Not all parameters listed in Table 2 are available for every experimental campaign, as data availability and level of detail vary depending on the specific objectives of each project. For more detailed descriptions, testing methodologies, and site-specific results, readers are referred to the original geotechnical reports included in the dataset repository.

### Wave overtopping detailed programs

The characteristics of incident waves and geometrical parameters used to generate the overtopping events are detailed in Table 3.

**Table 3:** Detailed wave events simulated at all test sites and characteristics of incident waves leading to levee overtopping reproduced by the wave overtopping simulator.

Test sections	Wave pattern acronym	t_st_ (hr)	Cotα	Cotβ	H_s_ (m)	q (l/s/m)	s_op_	T_p_ (s)	T_m_ (s)	N (/)	P_ov_ (%)	N_ov_ (/)
Delfzijl-BC	2m1lsm	6	4	3	2	1	0.04	5.7	4.7	4600	2.74	126
	2m5lsm	6	4	3	2	5	0.04	5.7	4.7	4600	10.38	477
	2m10lsm	6	4	3	2	10	0.04	5.7	4.7	4600	19.32	888
LLHPP -BC1-1	2m30lsm^a^	2	3	2.6	2	30	0.05	5.7	4.7	1532	10.44	160
LLHPP -BC1-2	2m30lsm^a^	2	3	2.6	2	30	0.05	5.7	4.7	1532	10.44	160
LLHPP -BC1-3	2m30lsm^a^	2	3	2.6	2	30	0.05	5.7	4.7	1532	11.75	180
LLHPP -BC2-1	0.5m60lsm	2	3	2.6	0.5	30	0.05	2.8	2.3	3129	45.3	1419
LLHPP -BC2-2	1m50lsm	2	3	2.6	1	30	0.05	4	3.3	2182	47.90	1045
LLHPP-LTCB1-A LLHPP-LTCB2-B LLHPP -LTCB3C	1m10lsm	2	3	2.6	1	10	0.05	4	3.3	2182	27	585
	1m30lsm^b^	2	3	2.6	1	30	0.05	4	3.3	2182	41	894^b^
	1m50lsm	2	3	2.6	1	50	0.05	4	3.3	2182	48	1045
LLHPP -LTCB2B LLHPP -LTCB3C	1m190lsm	4	3	2.6	1	190	0.05	4	3.3	4364	46	1991
	2m50lsm	2	3	2.6	2	50	0.04	5.7	4.7	1532	33	510
ERTL- WCC	1m1lsm	5	3	3	1	1	0.06	4	3.3	5455	4.13	225
	1m10lsm	5	3	3	1	10	0.06	4	3.3	5455	20.81	1135
	1m100lsm	5	3	3	1	100	0.06	4	3.3	5455	56.50	3082
	3.2m50lsm	6	4.5	3	3.2	50	0.04	8.2	6.8	3176	29.91	950
	3.2m100lsm^c^	6	4.5	3	3.2	100	0.04	8.2	6.8	3176	41.27	1311

^a^equivalent wave storm program based on Weibull distribution applied at all sections of the BC1; ^b^only the first 222 waves were released; ^c^instead of releasing the final sequence of waves (numbered 1101 to 1311 in the storm program), the initial sequence of waves (numbered 1 to 232) was simulated.

The parameters in Table 3 are defined as follows (from Eurotop 2018):

t_st_: storm event duration (hr).

Cotα: gradient of the dike slope on the waterside (−), where the angle α is measured between the dike face and a horizontal plane.

Cotβ: gradient of the dike slope on the landward side (−), where the angle β is measured between the dike face and a horizontal plane.

H_s_: significant wave height (m).

q: overtopping discharge (l/s/m).

s_op_: wave steepness (−).

T_m_: average wave period (s).

T_p_: peak wave period (s).

N: total number of incoming waves during the storm (−).

P_ov_: probability of overtopping (−).

N_ov_: number of overtopping waves during the storm (−).

**Fig. 19:**
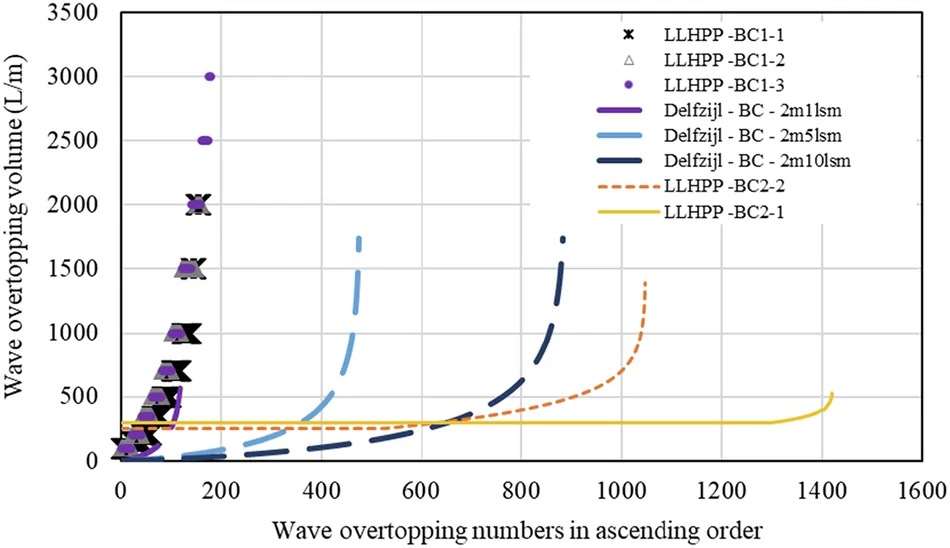
Distribution of wave overtopping volumes for different storm events as simulated by the Wave Overtopping Simulator (WOS) at LLHPP and Delfzijl on bare clay sections.

**Fig. 20:**
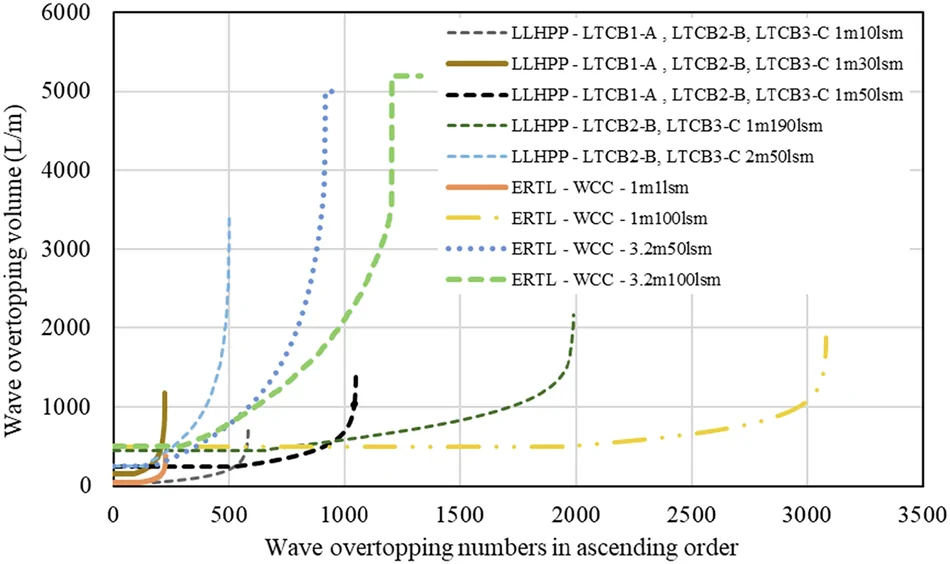
Distribution of wave overtopping volumes for different storm events as simulated by the Wave Overtopping Simulator (WOS) at LLHPP on lime-treated clay and ERTL on well compacted clay sections.

The overall wave overtopping programs executed across all the studies in this dataset are presented in Figs. 19, 20.

### Wavefront velocity data

The available data on wavefront velocity in different experimental campaigns are summarized in Table 4. For instance, the wavefront velocity was determined simultaneously using both manual analysis (Frame-by-Frame) and Wavefront tracker for a series of volumes in ERTL-WCC experiment.

**Table 4:** Data on the wavefront velocity from the field experiments.

Test Case	Recorded by	Footage	Manual analysis	Wavefront Tracker	Direct measurement
Delfzijl-BC	NA	NA	NA	NA	NA
LLHPP-BC	Drone	Available	Available	NA	NA
LLHPP-LTC	Fixed camera	Available	Available	NA	NA
ERTL-WCC	Drone	Available	Available	Available	NA

### EFA Laboratory experiments data

A summary of the EFA experimental program across the four test campaigns, including sample counts and sampling periods, is provided in Table 5.

**Table 5:** Available data on EFA test results and test specifications for the present dataset.

Test Case	Date of sampling	Sample Location	Number of core samples	Additional soil tests on samples	Comments
Delfzijl-BC	June 2023	Near the “Eneco recharge station” between Delfzijl and Termunterzijl	6	Particle size distribution, Initial water content, Atterberg limits	Cores 1, 2, and 3 were located approximately 60 meters away from cores 4, 5, and 6.
LLHPP-BC	February 2022	Next to the wave-overtopping test sections	5	Particle size distribution, Initial water content, Atterberg limits	Core sampling area spread over 30 m
LLHPP-LTC (Clay without additive)	NA	NA	NA	NA	No EFA test was performed for this section.
LLHPP-LTC (4% lime)	NA	NA	NA	NA	Only one EFA test was performed for this section. Due to limitations in repeatability, the result is not included in this database.
ERTL- WCC	NA	NA	NA	NA	No EFA test was performed for this section.

### Manual and SfM-photogrammetry data

Table 6 presents the monitoring techniques implemented in each experimental campaign to track erosion progression within the levee cover. For photogrammetry-based monitoring, the image acquisition system is also indicated. Time intervals and loading conditions associated with the manual measurements and photogrammetry processing are provided in the corresponding subfolders in the data repository, in the form of tables and graphs.

**Table 6:** Levee slope monitoring technique.

Test Case	SfM-Photogrammetry	Comments
Delfzijl-WCC	NA	Longitudinal profiles, as well as graphical products, including 0.5 m × 0.5 m raster image, derived from pointwise manual measurements are provided.
LLHPP-BC1	Available	Multiple fixed-camera system
LLHPP-BC2	Available	Multiple fixed-camera system
LLHPP-LTCB1	Available	One mobile camera
LLHPP-LTCB2	Available	One mobile camera
LLHPP-LTCB3	Available	One mobile camera
ERTL-WCC	Available	One mobile camera

## Technical Validation

### Application of close-range SfM-Photogrammetry

The accuracy of close-range SfM-Photogrammetry has previously been evaluated through comparison with terrestrial LiDAR scanners^29^. In addition, its reliability for erosion monitoring on levee slopes was validated using ground survey measurements in an earlier work^13^. For this purpose, randomly distributed points within selected test zones were measured with an RTK GNSS GPS system and compared with corresponding coordinates extracted from the 3D models (e.g., LLHPP-BC2-2 and LLHPP-LTCB1). This cross-validation confirmed that the technique produces high-resolution models with high positional accuracy for this type of application.

Further internal error analysis was performed directly within the photogrammetry software (Agisoft Metashape). By temporarily treating a subset of Ground Control Points (GCPs) as independent Check Points, the software recalculated their positions, enabling direct comparison between RTK-measured coordinates and photogrammetry-derived projections.

A limitation was observed when parts of the section were covered by shallow water (e.g., due to erosion scour hole formation), which can reduce surface reconstruction precision. This effect was minimized by allowing sufficient time for drainage prior to image acquisition. The photogrammetry software used (Agisoft Metashape) demonstrated robustness, consistently producing reliable models from different image sets acquired at the same time instant.

**Fig. 21:**
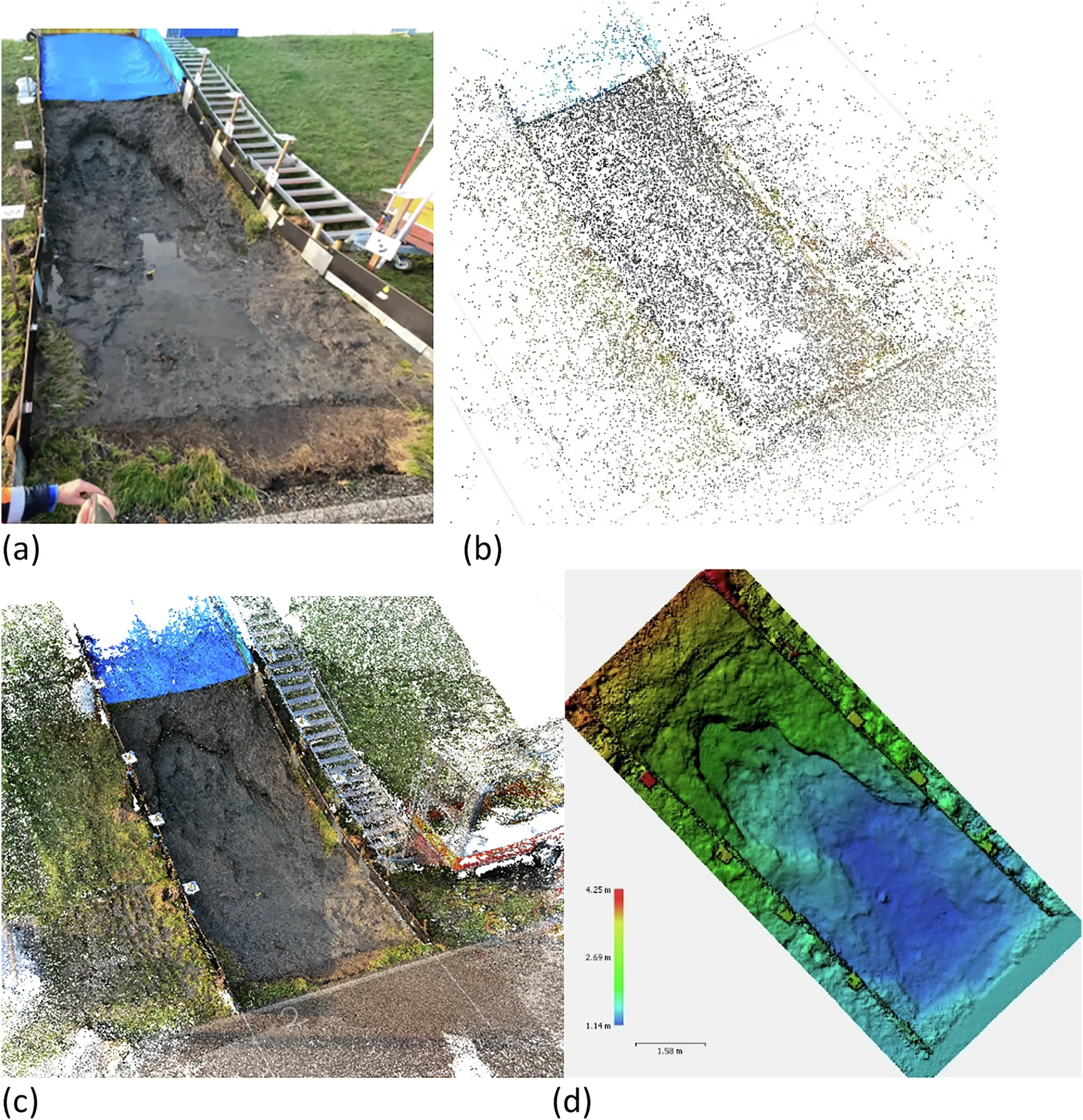
SfM-Photogrammetry procedure for 3D models reconstruction (**a**) Example of photographs, (**b**) photo alignment and resulted tie point model, (**c**) dense point cloud, (**d**) Digital elevation model (DEM) in case of ERTL-WCC at the end of experiment.

An overview of photogrammetric workflow used for the image processing and generating the DEMs is illustrated in Fig. 21(a–d).

As an example, some of these key steps applied to the image processing acquired using the mobile camera with an application to the ERTL-WCC test, are illustrated in Fig. 21(a–d).

The workflow involves image import (Fig. 21a), camera calibration (Fisheye camera), markers (GCP) detection, photo alignment (Fig. 21b), dense point cloud generation (Fig. 21c), georeferencing the GCPs, and DEMs generation (Fig. 21d).

Considering overall 15 Ground Control Points (GCPs) the average RMSE values are reported in Table 7 in X, Y, and Z directions. Potential error sources, such as light refraction caused by water presence and dark spots in the imagery are not reflected in these estimates.

**Table 7:** RMSE error estimates for DEM of ERTL-WCC at the end of experiment.

Error (RMSE)	X (mm)	Y (mm)	Z (mm)	XY (mm)	Total
Eight Ground Control Points (GCPs)	4.70	3.09	2.76	5.63	6.27
7 Check Points (CPs)	3.70	3.14	2.94	4.85	5.67

### Wavefront tracker

**Fig. 22:**
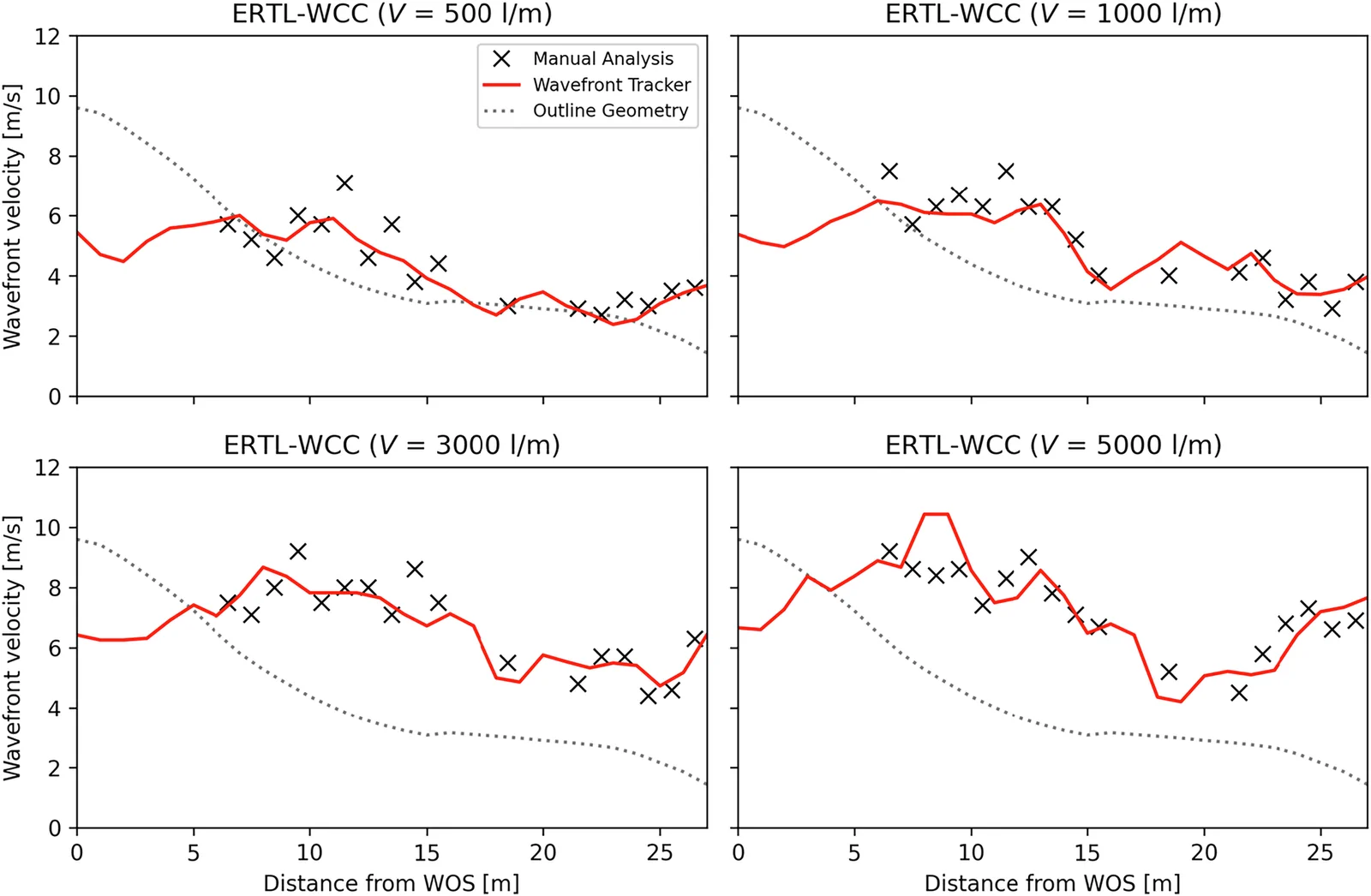
Comparison between manual analysis by Infram (black markers) and wavefront tracker (red line) for ERTL-WCC (outline geometry indicated by gray dotted line).

A comparison between the manual analysis and the Wavefront Tracker for ERTL-WCC is shown in Fig. 22. Both methods aim to detect the leading edge of the wavefront and generally produce similar results, with Standard Deviation of 1 m/s. These variations arise from differences in how the wavefront is detected. The Wavefront Tracker monitors multiple pixels along each meter mark (where the horizontal and vertical lines intersect in Fig. 15) and registers the wavefront once the brightness threshold is exceeded for any of these pixels. In contrast, the manual analysis visually determines when the wavefront crosses each meter mark. Slight discrepancies between the two methods can occur due to the precision of the manual assessment, the grid configuration, and the temporal (fps) and spatial resolution (m/px) of the video. For this study, the manual frame-by-frame analysis was conducted using video recorded at 60 fps, whereas the automated Wavefront Tracker utilized footage captured at 120 fps with a spatial resolution of 1920 × 1080.

## Usage Notes

The Python codes, along with usage examples, are provided within the corresponding data repository. The interpretation and contextual analysis of the DEMs, combined with hydraulic conditions, are included in a separate folder within the repository named post-processed. Users can refer to the examples in this folder to understand the potential downstream processing and interpretation of the raw data.

The database offers valuable insights in (1) improving the understanding of landward erosion mechanisms on the landward slope of levees under wave overtopping, particularly in relation to soil properties; (2) highlighting the influence of hydraulic loading and potential scale effects between laboratory and field conditions (3) enhancing soil erodibility testing techniques and erosion modelling tools, and (4) enabling more generalized interpretation of soil erodibility and translation of the findings to other levees with similar configurations.

While this database offers comprehensive insights, users should be aware of the scope and limitations of the data. The experimental conditions primarily reflect the erosion resistance of “bare clay” (where the 20-cm-thick topsoil was removed, as seen in the Delfzijl and LLHPP-BC campaigns). While the structure aging and good grass quality coverage allowed for the development of mature root systems prior to topsoil removal, quantitative measurements of these root parameters are not available for these campaigns.

On the other hand, “newly placed clay,” such as in the LLHPP-LTC and ERTL-WCC campaigns, had a short establishment period insufficient for partial or full root network development. Consequently, these datasets should be used with caution when assessing levees with “long-term vegetation cover” or significant “bioturbation,” as mature root systems and biological activity can substantially alter soil erodibility and structural integrity. Furthermore, the transferability of the data to other locations may be influenced by the specific environmental conditions present during the field measurements. Factors such as temperature and moisture content, which is strongly correlated with the shear strength of the soil, can significantly affect the levee’s response to hydraulic loading. Users are encouraged to carefully consider these site-specific and temporal variables when applying the dataset for model calibration or flood defence design in different geographic or climatic contexts.

## Data Availability

All photogrammetric processing was performed using the commercial software Agisoft Metashape Professional (Agisoft LLC, Version 1.8). The software and its documentation are available from the developer at: Agisoft LLC. (2022). Agisoft Metashape User Manual: Professional Edition (Version 1.8). Access to the software is subject to standard licensing restrictions. Custom Python codes were developed for two purposes: to facilitate the visualization of measured data during the EFA tests and to aid in the visualization and analysis of the Digital Elevation Models (DEMs). These codes, along with usage examples, are freely available and provided within the corresponding data repository. Furthermore, the Wavefront Tracker Python source code used in this study to automate wavefront velocity detection from video footage is publicly available on GitHub at https://github.com/nielsvandervegt/wavefront_tracker. The repository includes documentation to assist future researchers in applying the tracking methodology to their own video datasets. Finally, the data record associated with this workis stored at 10.14428/DVN/59TYSY (2026). Readers are encouraged to contact the corresponding authors with any additional queries.
